# Calcium-sensing receptors signal constitutive macropinocytosis and facilitate the uptake of NOD2 ligands in macrophages

**DOI:** 10.1038/ncomms11284

**Published:** 2016-04-06

**Authors:** Johnathan Canton, Daniel Schlam, Christian Breuer, Michael Gütschow, Michael Glogauer, Sergio Grinstein

**Affiliations:** 1 Program in Cell Biology, Hospital for Sick Children, 686 Bay Street, Toronto, Ontario, Canada M5G 0A4; 2 Pharmaceutical Institute, Pharmaceutical Chemistry I, University of Bonn, An der Immenburg 4, D-53121 Bonn, Germany; 3 Faculty of Dentistry, University of Toronto, 150 College Street, Toronto, Ontario, Canada M5G 1G6; 4 Keenan Research Centre of the Li Ka Shing Knowledge Institute, St Michael's Hospital, 290 Victoria Street, Toronto, Ontario, Canada M5C 1N8

## Abstract

Macropinocytosis can be induced in several cell types by stimulation with growth factors. In selected cell types, notably macrophages and dendritic cells, macropinocytosis occurs constitutively, supporting the uptake of antigens for subsequent presentation. Despite their different mode of initiation and contrasting physiological roles, it is tacitly assumed that both types of macropinocytosis are mechanistically identical. We report that constitutive macropinocytosis is stringently calcium dependent, while stimulus-induced macropinocytosis is not. Extracellular calcium is sensed by G-protein-coupled calcium-sensing receptors (CaSR) that signal macropinocytosis through Gα-, phosphatidylinositol 3-kinase and phospholipase C. These pathways promote the recruitment of exchange factors that stimulate Rac and/or Cdc42, driving actin-dependent formation of ruffles and macropinosomes. In addition, the heterologous expression of CaSR in HEK293 cells confers on them the ability to perform constitutive macropinocytosis. Finally, we show that CaSR-induced constitutive macropinocytosis facilitates the sentinel function of macrophages, promoting the efficient delivery of ligands to cytosolic pattern-recognition receptors.

## Abstract (web summary)

Macropinocytosis can be induced in several cell types by growth factors to promote nutrient acquisition. Here the authors find that constitutive macropinocytosis, unique to dendritic cells and macrophages, requires the activity of calcium-sensing receptors.

## Graphical abstract

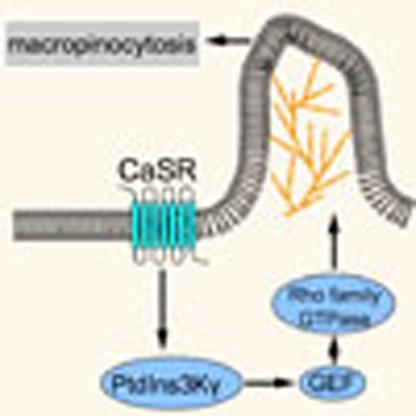

Macropinocytosis is an actin-driven process whereby cells internalize large volumes of extracellular fluid, generating phase-bright vacuoles (>250 nm). Many cell types generate such vacuoles, known as macropinosomes, in response to growth factor stimulation. In these instances, macropinocytosis is intended for nutrient acquisition, representing a major amino-acid supply route that enables cell growth^1^. Other proposed functions include recycling of adhesion receptors to the leading edge of migratory cells^2^, bulk membrane retrieval^3^ and growth cone collapse in nerve cells^4^.

To trigger macropinocytosis, growth factors remodel the lipid microenvironment. Phosphatidylinositol 4,5-*bis*phosphate (PtdIns(4,5)P_2_) is converted to phosphatidylinositol 3,4,5-*tris*phosphate (PtdIns(3,4,5)P_3_) and in parallel also hydrolysed into diacylglycerol (DAG) and inositol 3,4,5-*tris*phosphate (Ins(3,4,5)P_3_) by phospholipase Cγ (PLCγ)^5^,^6^,^7^. The subsequent conversion of DAG to phosphatidic acid (PtdOH) by diacylglycerol kinase and the sequential breakdown of PtdIns(3,4,5)P_3_ into phosphatidylinositol 3,4-*bis*phosphate and finally phosphatidylinositol 3-phosphate are required for the completion of macropinocytosis^7^,^8^.

The spatially and temporally restricted changes in lipid composition contribute to the recruitment of several protein effectors, including guanine nucleotide exchange factors (GEFs) that control the activity of Rho-family GTPases. One such GEF, T-cell lymphoma invasion and metastasis-inducing protein 1 (TIAM1), has both a PtdIns(3,4,5)P_3_-binding PH domain and a PtdOH-interacting polybasic domain that mediate its recruitment to nascent ruffles^8^,^9^. The consequent activation of Rac1 promotes the formation of branched actin networks that drive membrane extensions and macropinocytic cup formation. Other Rho family GTPases, such as Cdc42 and RhoG, are also required for macropinocytosis, although details of their function remain less clear^10^,^11^.

Although macropinocytosis can be induced in a variety of cell types by growth factors and other stimuli, dendritic cells (DCs) and macrophages share a unique behaviour: they perform macropinocytosis constitutively. This intrinsic process is astonishingly active: macrophages were estimated to internalize the equivalent of their entire cell surface every 33 min (ref. 12), while human DCs take up >1,000 μm (ref. 3) of extracellular fluid—roughly 40% of the volume of the cell—every hour^13^. This feature allows for the continuous capture of antigens, which are processed and presented to the adaptive immune system. Despite their different mode of initiation and contrasting physiological roles, it is tacitly assumed that stimulus induced and constitutive macropinocytosis are mechanistically identical. To date, however, the molecular mechanisms underlying these two modes have not been formally compared. Here we identify key differences between constitutive and growth factor-induced macropinocytosis in primary phagocytes. In particular, a major distinguishing feature—the requirement for extracellular calcium—is studied in detail and reveals the involvement of calcium-sensing receptors in constitutive macropinocytosis.

## Results

### Macrophages perform distinct modes of macropinocytosis

**Figure 1:**
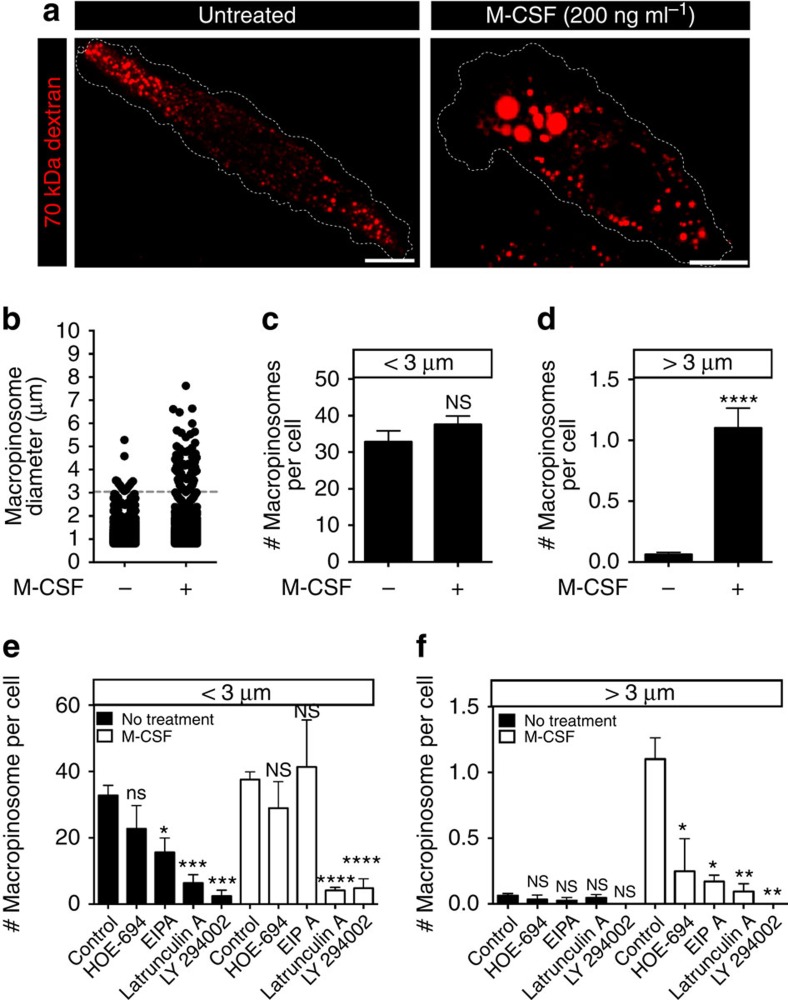
Two distinct modes of macropinocytosis exist in hMDMs. (**a**) hMDMs were incubated with fluorescently labelled 70 kDa dextran (0.025 mg ml^−1^) for 15 min at 37 °C, in either the presence or absence of M-CSF (200 ng ml^−1^). Cells were then washed and imaged immediately by spinning disc confocal microscopy. (**b**) Macropinosomes were then counted using ImageJ software and the diameter of each macropinosome was recorded and plotted. A size cutoff (dashed line) was set to distinguish between constitutive and growth factor-induced macropinosomes. The number of macropinosomes under (**c**) or above (**d**) 3 μm in diameter per cell was plotted. hMDMs were pretreated with HOE-694 (10 μM), EIPA (10 μM), latrunculin A (2 μM) or LY294002 (10 μM) for 1 h and then incubated with fluorescently labelled 70 kDa dextran (0.025 mg ml^−1^) for 15 min at 37 °C, in either the presence or absence of M-CSF (200 ng ml^−1^). The number of macropinosomes under (**e**) or above (**f**) 3 μm in diameter is plotted. Data represent the means±s.e.m. of at least three independent experiments using cells from at least 2 separate healthy donors. Scale bar, 10 μm. *P≤0.05, **P≤0.01, ***P≤0.001, ****P≤0.0001, NS, not significantly different.

Unstimulated DCs and macrophages undergo constitutive ruffling^14^. Convergence and closure of ruffles to form large cytosolic vacuoles has been documented in both cell types^8^,^10^. We explored whether this mode of constitutive macropinocytosis differs from that induced by growth factors. Large (70 kDa) dextran was used for our assays as it has been shown to be internalized exclusively via macropinocytosis, and not by other modes of endocytosis such as clathrin-coated pits or caveolae^15^. To ensure that this was indeed the case in myeloid cells, we compared the uptake of the large dextran by primary human monocyte-derived macrophages (hMDMs) that were either untreated, or were pretreated with latrunculin A (an actin disruptor) or with *Clostridium difficile* toxin B (an inhibitor of small Rho GTPases), agents shown earlier to block macropinocytosis^16^,^17^. The uptake of 70 kDa dextran, which is patent in unstimulated hMDMs (Fig. 1; Supplementary Figs 1 and 2a) was obliterated by either latrunculin or *C. difficile* toxin B (Supplementary Fig. 2a). Under these conditions, uptake of transferrin—a canonical clathrin-dependent cargo^18^—proceeded normally, and the distribution of early (Rab5-positive) and late (LAMP1-positive) endosomal compartments was unaffected (Supplementary Fig. 2a–c). These observations validate the use of 70 kDa dextran to monitor macropinocytosis in macrophages.

We proceeded to compare constitutive and growth factor-stimulated macropinocytosis. To analyse constitutive macropinocytosis hMDMs were bathed in serum-free medium for the duration of the dextran uptake period (15 min; similar results were obtained when the cells were, in addition, preincubated in the absence of serum for up to 16 h). hMDMs formed numerous cytosolic vacuoles containing 70 kDa dextran, whether incubated in the presence or absence of macrophage colony-stimulating factor (M-CSF; Fig. 1a). The size of the resultant macropinosomes was, however, different under both conditions: vacuoles larger than 3 μm were often formed when M-CSF was present, while smaller macropinosomes were generated in its absence (Fig. 1a). We applied a lower size cutoff of 0.75 μm to exclude endosomes and set an arbitrary size threshold at 3 μm to distinguish between the two forms of macropinocytosis (Fig. 1b). Note that macropinosomes smaller than 3 μm were observed under both conditions and their frequency was unaltered by M-CSF. In contrast, the number of macropinosomes larger than 3 μm was negligible in the absence of M-CSF, but increased on exposure to the growth factor (Fig. 1c,d). These observations suggest that the thresholding approach appropriately distinguished between macropinosomes formed constitutively and those induced by M-CSF. While the 15 min allowed for dextran uptake yielded large and reliable signals, it was conceivable that the occurrence of fusion or fission events during this period made the quantification of macropinosomes inaccurate. However, we found that a much shorter uptake period (3 min) yielded very similar results (Supplementary Fig. 1a–d). Moreover, we monitored macropinosomes loaded by 1-min incubation with 70 kDa dextran for the subsequent 15 min and found that while their size decreased and fluorescence intensity increased—likely as a consequence of fission of small vesicles devoid of dextran—their number was not significantly altered (Supplementary Fig. 1e,f), and that they mix minimally with the late endosomal compartment (Supplementary Movie 1). Altogether, these findings validate the accuracy of the method used to quantify macropinosome formation.

We next confirmed that, under the conditions described above, the vacuoles containing dextran were *bona fide* macropinosomes. Macropinocytosis is typically defined as an amiloride sensitive, phosphatidylinositol 3-kinase (PtdIns3K)- and actin-dependent process^19^. Using the amiloride derivatives HOE-694 and 5-(*N*-ethyl-*N*-isopropyl)amiloride (EIPA), the PtdIns3K inhibitor LY294002 and the actin-disrupting agent latrunculin A, we assessed whether the vacuoles formed fulfilled the canonical definition of macropinosomes. Both the constitutive and stimulated forms of macropinocytosis were sensitive to the drugs tested, with the exception that the smaller (<3 μm) vacuoles were less sensitive to the amiloride derivatives than anticipated (Fig. 1e,f). The latter observation is reminiscent of earlier reports where the constitutive uptake of fluid by immature DCs was found to be similarly unaffected by amiloride derivatives^20^. Altogether, these observations establish that there are two morphologically distinct modes of macropinocytosis in hMDMs and that these can be further distinguished by their sensitivity to amiloride derivatives.

### Constitutive macropinocytosis requires extracellular Ca^2+^

**Figure 2:**
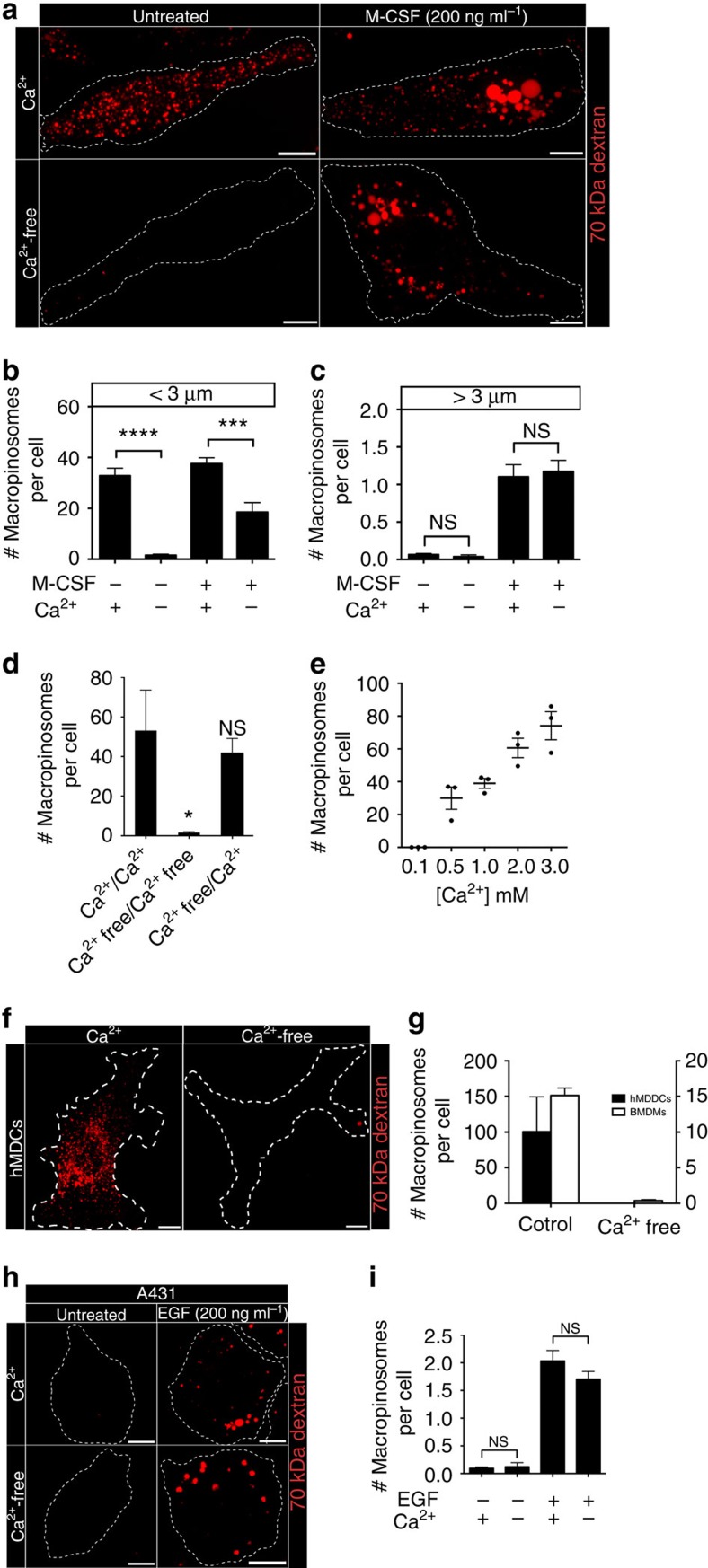
Removal of extracellular calcium abolishes constitutive, but not growth factor-induced macropinocytosis. (**a**) hMDMs were incubated with fluorescently labelled 70 kDa dextran (0.025 mg ml^−1^) for 15 min at 37 °C, in the presence or absence of M-CSF (200 ng ml^−1^), in either calcium-containing or calcium-free medium (see Methods). The number of macropinosomes under (**b**) or above (**c**) 3 μm in diameter is plotted. (**d**) hMDMs were preincubated in either calcium-containing or calcium-free medium and then allowed to undergo macropinocytosis for 15 min at 37 °C. The total number of macropinosomes per cell is plotted. (**e**) hMDMs were incubated with fluorescently labelled 70 kDa dextran (0.025 mg ml^−1^) for 15 min at 37 °C in Na^+^-rich buffer containing the indicated concentrations of CaCl_2_. The total number of macropinosomes per cell is plotted. (**f**) hMDDCs were incubated with fluorescently labelled 70 kDa dextran (0.025 mg ml^−1^) for 15 min at 37 °C, in either calcium-containing or calcium-free medium. (**g**) hMDDCs and murine BMDMs were incubated with fluorescently labelled 70 kDa dextran (0.025 mg ml^−1^) for 15 min at 37 °C, in either calcium-containing or calcium-free medium. The total number of macropinosomes per cell is plotted. (**h**) A431 cells were incubated with fluorescently labelled 70 kDa dextran (0.025 mg ml^−1^) for 15 min at 37 °C, in the presence or absence of EGF (200 ng ml^−1^), in either calcium-containing or calcium-free medium. (**i**) The total number of macropinosomes per cell is plotted for each condition, as indicated. Data represent the means±s.e.m. of at least three independent experiments using cells from at least two separate healthy donors. Scale bar, 10 μm. *P≤0.05, ***P≤0.001, ****P≤0.0001, NS, not significantly different.

Our results suggest that macropinocytosis is more heterogeneous than previously appreciated. In this regard it resembles phagocytosis, which encompasses a variety of processes that while sharing some gross phenotypic properties, differ in their molecular mechanism. For instance, some forms of phagocytosis require calcium while others do not^21^. We used this criterion to establish whether the constitutive and M-CSF-induced forms of macropinocytosis are distinct. It is worth noting that calcium is required for optimal M-CSF signalling^22^. Strikingly, and contrary to our expectation, removal of extracellular calcium abolished the constitutive form of macropinocytosis, but did not affect M-CSF-induced macropinocytosis (Fig. 2a–c; Supplementary Fig. 1a–d). Similar results were obtained when, instead of counting the number of macropinosomes formed, the quantity of 70 kDa dextran taken up was assessed by flow cytometry (Supplementary Fig. 3a–c). The effect was reversible, as the readdition of calcium to cells in calcium-free medium restored constitutive macropinocytosis (Fig. 2d). By contrast, removal of extracellular calcium did not affect the uptake of transferrin, nor the distribution of Rab5 and LAMP1, indicating that other forms of endocytosis remained unaltered (Supplementary Fig. 2a–c).

The preceding results imply that constitutive macropinocytosis is a calcium-dependent event; indeed, the rate of macropinocytosis was directly correlated to the concentration of extracellular calcium (Fig. 2e). To ensure that this feature is common to phagocytes from other species, we tested murine bone marrow-derived macrophages (mBMDMs); these cells proved to be equally sensitive to omission of calcium (Supplementary Fig. 3d; Fig. 2g), as were human monocyte-derived dendritic cells (hMDDCs; Fig. 2f,g), which also perform macropinocytosis constitutively^8^,^20^. We also tested A431 cells, an epidermal cell line that expresses high levels of the epidermal growth factor (EGF) receptor. In these cells macropinocytosis is negligible in the absence of growth factors, but it is greatly stimulated by addition of EGF (Fig. 2h,i). Removal of calcium had no effect on the induction of macropinocytosis by EGF (Fig. 2h,i). Altogether these data demonstrate that the constitutive form of macropinocytosis in phagocytes requires extracellular calcium; by contrast, growth factor-induced macropinocytosis is calcium independent.

### [Ca^2+^]_*i*_ does not affect constitutive macropinocytosis

**Figure 3:**
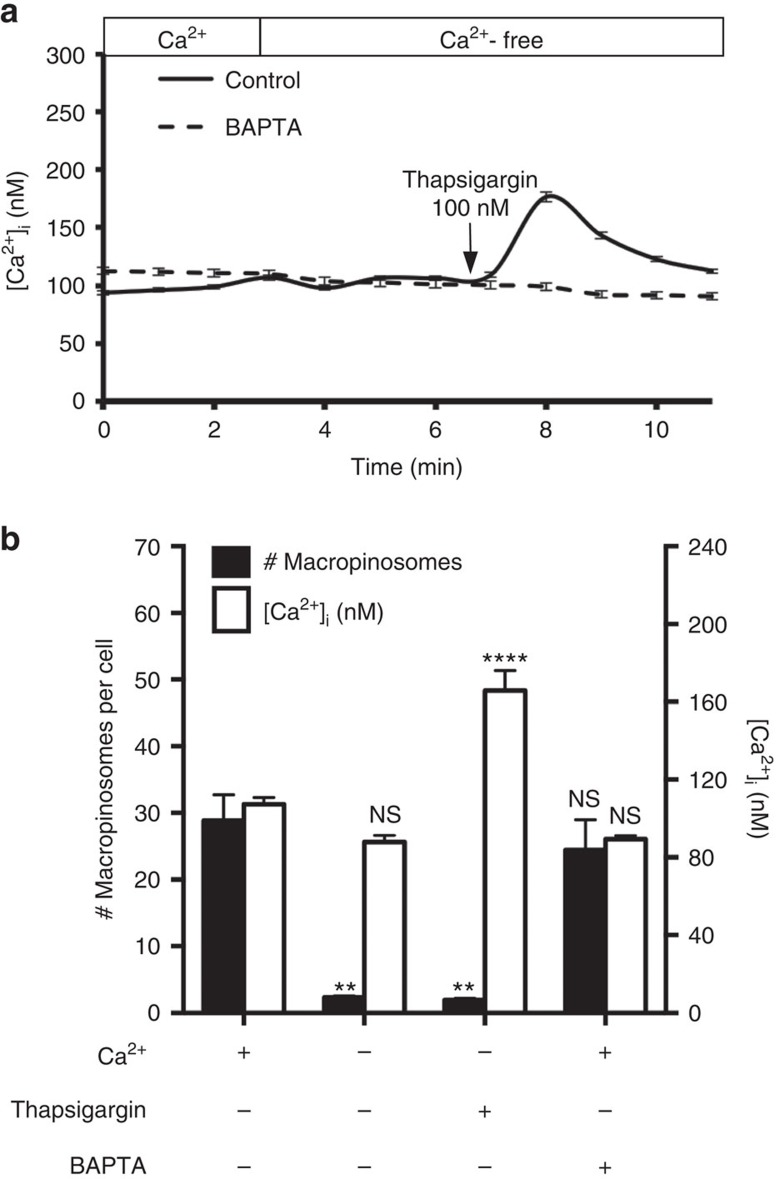
Role of intracellular calcium in constitutive macropinocytosis. (**a**) hMDMs were loaded with 3 μM Fura2-AM and, where indicated, with 10 μM BAPTA-AM (see Methods), and imaged for 3 min in calcium-containing medium, acquiring an image every minute. The medium was then replaced with calcium-free medium and the cells imaged for an additional 4 min. Thapsigargin (100 nM) was then added and images were acquired for an additional 5 min. Data are representative of at least 30 independent determinations±s.e.m. (**b**) hMDMs were preloaded with Fura2-AM and treated with or without thapsigargin (100 nM), BAPTA-AM (10 μM), and extracellular calcium as indicated. The total number of macropinosomes per cell and the corresponding [Ca^2+^]_I_ were then plotted. Data represent the means±s.e.m. of at least three independent experiments using cells from at least two separate healthy donors. **P≤0.01, ****P≤0.0001, NS, not significantly different.

Influx of extracellular calcium is an essential component of cytosolic calcium ([Ca^2+^]_*i*_) homeostasis. Removal of external calcium eliminates [Ca^2+^]_*i*_ transients that result from influx across the plasmalemma, and causes a progressive decrease in the steady state [Ca^2+^]_*i*_. Alterations in [Ca^2+^]_*i*_, a well acknowledged second messenger, can conceivably account for the impairment of macropinocytosis observed when external calcium was omitted. Indeed, a variety of Ca^2+^-dependent effectors, including myosin light-chain kinase^23^,^24^, protein kinase C^5^,^25^ and phospholipase C (PLC)^26^,^27^, are involved in both macropinocytosis and phagocytosis. To determine whether the effect of removing extracellular calcium on constitutive macropinocytosis was due to changes in [Ca^2+^]_*i*_, we monitored [Ca^2+^]_*i*_ and the efficiency of macropinocytosis simultaneously; [Ca^2+^]_*i*_ was measured by ratio imaging using Fura2. The removal of extracellular calcium had a minimal effect on [Ca^2+^]_*i*_ within a timeframe relevant to the macropinocytosis uptake assays: [Ca^2+^]_*i*_ was not significantly different when measured before and shortly (≈30 s) after external calcium removal (Fig. 3a,b). Even when measured after 15 min, the end point of the macropinocytosis determinations, [Ca^2+^]_*i*_ had decreased by only 19 nM, from 107±4 nM to 88±3 nM (means±s.e. of 28 determinations; Supplementary Fig. 4a). Under these conditions, constitutive macropinocytosis was virtually abolished (Fig. 3b). We used thapsigargin to rule out the possibility that the minute change in basal [Ca^2+^]_*i*_ that develops during the course of the assay was responsible for the inhibition of macropinocytosis. By inhibiting the Ca^2+^-ATPase, thapsigargin causes net release of calcium from the endoplasmic reticulum. In cells bathed in calcium-free medium, this is manifested as an increase in [Ca^2+^]_*i*_ (Fig. 3a and Supplementary Fig. 4b). While the increase is transient, it nevertheless suffices to maintain [Ca^2+^]_*i*_ above the baseline level throughout the macropinocytosis assay. This enabled us to test the effects of external calcium removal under conditions where [Ca^2+^]_*i*_ did not decrease. As shown in Fig. 3b, macropinocytosis was nevertheless obliterated. These findings imply that the inhibitory effect of calcium removal is not attributable to decreased [Ca^2+^]_*i*_, and suggest that [Ca^2+^]_*i*_ transients do not suffice to induce macropinocytosis.

It was conceivable that juxtamembrane rapid [Ca^2+^]_*i*_ transients, not detectable by global measurements of [Ca^2+^]_*i*_ were responsible for macropinocytosis. We used 1,2-*bis* (2-aminophenoxy)ethane-*N*,*N*,*N*′,*N*′-tetraacetic acid (BAPTA), a fast calcium chelator, to evaluate this possibility. We loaded cells with BAPTA and confirmed that the chelator effectively buffered [Ca^2+^]_*i*_ transients. As illustrated in Fig. 3a, the [Ca^2+^]_*i*_ elevation normally induced by thapsigargin was eliminated when cells were preloaded with BAPTA. Importantly, buffering [Ca^2+^]_*i*_ with BAPTA had no effect on constitutive macropinocytosis (Fig. 3b). These experiments demonstrate that the inhibition of constitutive macropinocytosis associated with external calcium removal was not caused by elimination of [Ca^2+^]_*i*_ transients or by reduction in the resting level of [Ca^2+^]_*i*_. Instead, extracellular calcium appears to be exerting its effect on the outer surface of the cell.

### CaSR is necessary for constitutive macropinocytosis

**Figure 4:**
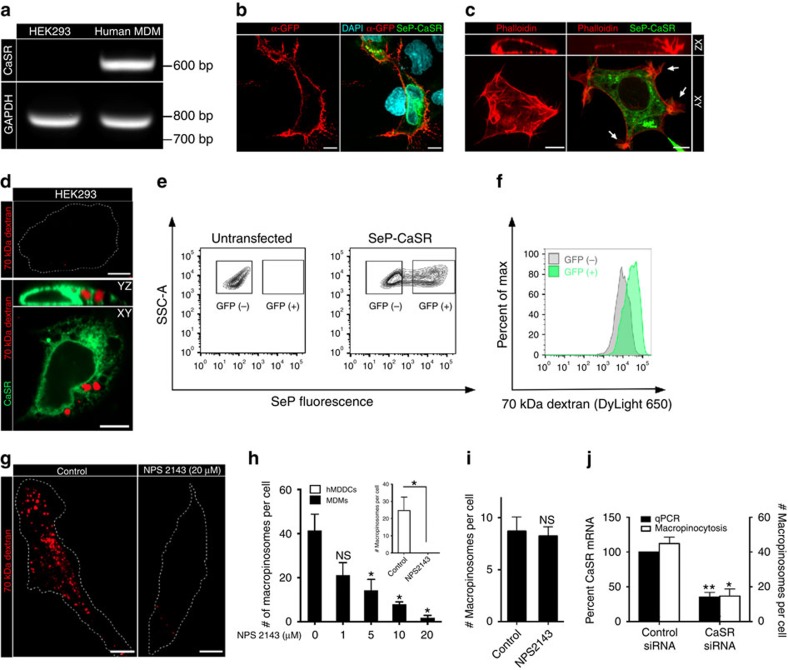
CaSR is necessary for constitutive macropinocytosis in hMDMs and is sufficient to induce macropinocytosis when heterologously expressed in HEK293 cells. (**a**) The expression of CaSR mRNA in HEK293 cells and hMDMs was determined by RT–PCR. The expression of GAPDH mRNA was determined as a control. (**b**) HEK293 cells were transfected with SeP-CaSR and, 24 h after transfection, were incubated on ice with an anti-GFP monoclonal antibody for 15 min to label SeP-CaSR expressed at the cell surface. Cells were then washed, fixed and labelled with a fluorescent secondary antibody (red). Cell nuclei were labelled with DAPI (cyan). (**c**) HEK293 cells were transfected with SeP-CaSR and, 24 h after transfection, were labelled with fluorescent phalloidin (red). (**d**) Untransfected (upper panel) and HEK293 cells transfected with SeP-CaSR (lower panel) were incubated with labelled 70 kDa dextran (0.025 mg ml^−1^) for 15 min at 37 °C. Cells were then imaged to quantify the uptake of dextran from the fluid phase. (**e**,**f**) Untransfected and HEK293 cells transfected with SeP-CaSR were incubated with DyLight 650-labelled 70 kDa dextran (0.025 mg ml^−1^) for 15 min at 37 °C. Cells were then gently lifted and dextran uptake assessed by flow cytometry. Gates were drawn on GFP(+) (SeP-positive) and GFP(-) (SeP-negative) populations as shown in (**e**) and the relative dextran uptake in the respective populations in shown in (**f**). (**g**) hMDMs were pretreated with the indicated dose of the specific CaSR antagonist NPS2143 and then incubated with labelled 70 kDa dextran (0.025 mg ml^−1^) for 15 min at 37 °C. (**h**) The total number of macropinosomes per cell is plotted at the indicated concentration of NPS2143 for hMDMs (main panel) and hMDDCs (inset, where 10 μM was used). (**i**) hMDMs were pretreated with NPS2143 (10 μM), and then incubated with 70 kDa dextran (0.025 mg/ml) and M-CSF (200 ng/mL) for 15 min at 37 °C. (**j**) CaSR expression was knocked down in hMDMs using CaSR-specific siRNA oligonucleotides. The cells were then incubated with labelled 70 kDa dextran (0.025 mg ml^−1^) for 15 min at 37 °C. The expression level of CaSR mRNA, normalized to actin mRNA, was determined by quantitative PCR (qPCR); the total number of macropinosomes per cell is also plotted. Scale bar, 10 μm. *P≤0.05, **P≤0.01, NS, not significantly different.

In specialized cell types, the level of extracellular calcium is monitored by a G-protein-coupled receptor, CaSR. CaSR is expressed in myeloid cells, where it has been implicated in the activation of the inflammasome pathway^28^. We therefore considered the possibility that CaSR was involved in constitutive macropinocytosis. We initially confirmed that CaSR is expressed in macrophages: unlike HEK293, which were shown earlier to lack CaSR^29^, transcript encoding the receptor was readily detectable by RT–PCR in hMDMs (Fig. 4a). We next investigated whether activation of CaSR sufficed to elicit macropinocytosis. To this end we used HEK293 cells, which lack the receptor and do not normally perform macropinocytosis (top panel, Fig. 4d). We first confirmed that a fraction of the heterologously expressed CaSR was able to traffic to the plasma membrane in these cells. A CaSR fusion protein containing an extracellular superecliptic phluorin (SeP) tag was readily detected on the surface of non-permeabilized HEK293 cells using an anti-GFP antibody (Fig. 4b). Strikingly, heterologous expression of CaSR not only induced vigorous ruffling in HEK293 cells, as reported^29^, but sufficed to induce constitutive macropinocytosis (Fig. 4c,d). Using flow cytometry we determined that CaSR-expressing cells accumulated 70 kDa dextran preferentially, compared to untransfected HEK293 cells (Fig. 4e,f). As anticipated, the constitutive macropinocytosis of CaSR-expressing HEK293 cells was reduced by removal of extracellular calcium (Supplementary Fig. 5a).

That CaSR is in fact responsible for constitutive macropinocytosis in myeloid cells was verified using NPS2143, a selective antagonist of the receptor^30^. Exposure of hMDMS to NPS2143 resulted in a dose-dependent inhibition of constitutive macropinocytosis (Fig. 4g,h), accompanied by elimination of the spontaneous ruffling (Supplementary Movie 2 and 3). NPS2143 also potently inhibited constitutive macropinocytosis in hMDDCs (Fig. 4h, inset). Similar results were obtained with a second, structurally unrelated CaSR inhibitor, namely Calhex 231, which was somewhat less effective than NPS2143 (Supplementary Fig. 5b). As with the removal of external calcium, NPS2143 treatment did not alter the uptake of transferrin or the distribution of Rab5 and LAMP1, indicating that other forms of endocytosis were unaffected (Supplementary Fig. 2a–c). Last and consistent with the findings made when external calcium was removed, exposure of hMDMs to NPS2143 did not affect growth factor-induced macropinocytosis (Fig. 4i).

Although suggestive, this pharmacological evidence is not conclusive, due to possible off-target effects of these drugs. More definitive evidence of the involvement of CaSR in constitutive macropinocytosis was obtained by silencing the expression of the receptors with siRNA. Using electroporation, we achieved a ≈70% reduction in the levels of CaSR transcript, as determined by quantitative PCR, in hMDMs (Fig. 4j). This was accompanied by a commensurate reduction in the macropinocytic efficiency (Fig. 4j). Collectively, these experiments demonstrate that CaSR is required for constitutive macropinocytosis in macrophages.

### CaSR signals constitutive macropinocytosis via Gα

**Figure 5:**
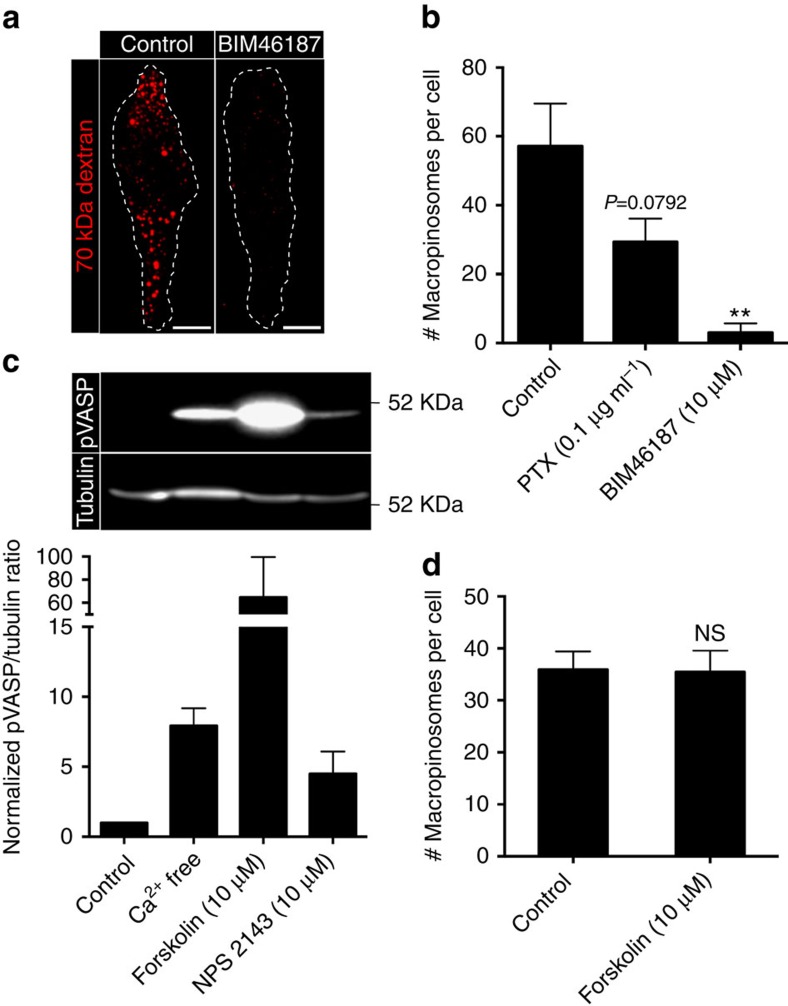
Gα_*i*_-dependent signalling is required for constitutive macropinocytosis. (**a**) hMDMs were pretreated in the absence or presence of BIM46187 (10 μM) for 1 h and then incubated with fluorescent 70 kDa dextran (0.025 mg ml^−1^) for 15 min at 37 °C. (**b**) hMDMs were pretreated with BIM46187 (10 μM) or PTX (0.1 μg ml^−1^) for 1 h and then incubated with fluorescent 70 kDa dextran (0.025 mg ml^−1^) for 15 min at 37 °C. The total number of macropinosomes per cell is plotted. (**c**) hMDMs were incubated with forskolin (10 μM) or NPS2143 (10 μM) in either calcium-containing or calcium-free medium for 30 min, then lysed, fractionated by 12% SDS–PAGE and subjected to immunoblotting with antibodies to phosphorylated VASP (pVASP) and α-tubulin (as loading control). A representative immunoblot is shown on top and quantification of the pVASP/α-tubulin ratio from three independent experiments is shown below. (**d**) hMDMs were pretreated with forskolin (10 μM) for 30 min and then incubated with labelled 70 kDa dextran (0.025 mg ml^−1^) for 15 min at 37 °C. The total number of macropinosomes per cell was then plotted. Scale bar, 10 μm. **P≤0.01, NS, not significantly different.

Like other serpentine receptors, CaSR couples to heterotrimeric G proteins. To assess the involvement of G proteins in macropinocytosis we used the recently described pan-Gα inhibitor, BIM46187 (refs 31, 32), and the Gα_*i*_ inhibitor, pertussis toxin (PTX). BIM46187 potently inhibited constitutive macropinocytosis, indicating a requirement for Gα-dependent signalling (Fig. 5a,b). PTX also inhibited macropinocytosis, albeit to a lesser extent, suggesting that Gα_*i*_-dependent signalling is at least partially involved (Fig. 5b). The observed inhibition could have resulted from suppression of a stimulatory signal, or from the generation of an inhibitory signal. Cytosolic 3′-5′-cyclic AMP (cAMP) levels are controlled by both stimulatory (Gα_*s*_) and inhibitory (Gα_*i*_) G proteins, and inhibition of the latter is anticipated to elevate cAMP. Increased cAMP could conceivably account for the observations, because it controls the phosphorylation and activity of vasodilator-stimulated phosphoprotein (VASP), a regulator of actin filament elongation; of note, VASP has been implicated in the reorganization of the actin cytoskeleton that underlies macropinocytosis^33^. Indeed, we found that removal of calcium or inhibition of CaSR by NPS2143 increased the phosphorylation of VASP, likely due to stimulation of protein kinase A activity in response to elevated cAMP^34^ (Fig. 5c). However, an even greater increase in VASP phosphorylation, induced by direct stimulation of adenylyl cyclase with forskolin, had no effect on constitutive macropinocytosis (Fig. 5d). These observations imply that the inhibitory effects of BIM46187 and PTX on macropinocytosis are not a consequence of the increase in cAMP.

### CaSR signals macropinocytosis via PtdIns3Kγ and PLCγ

**Figure 6:**
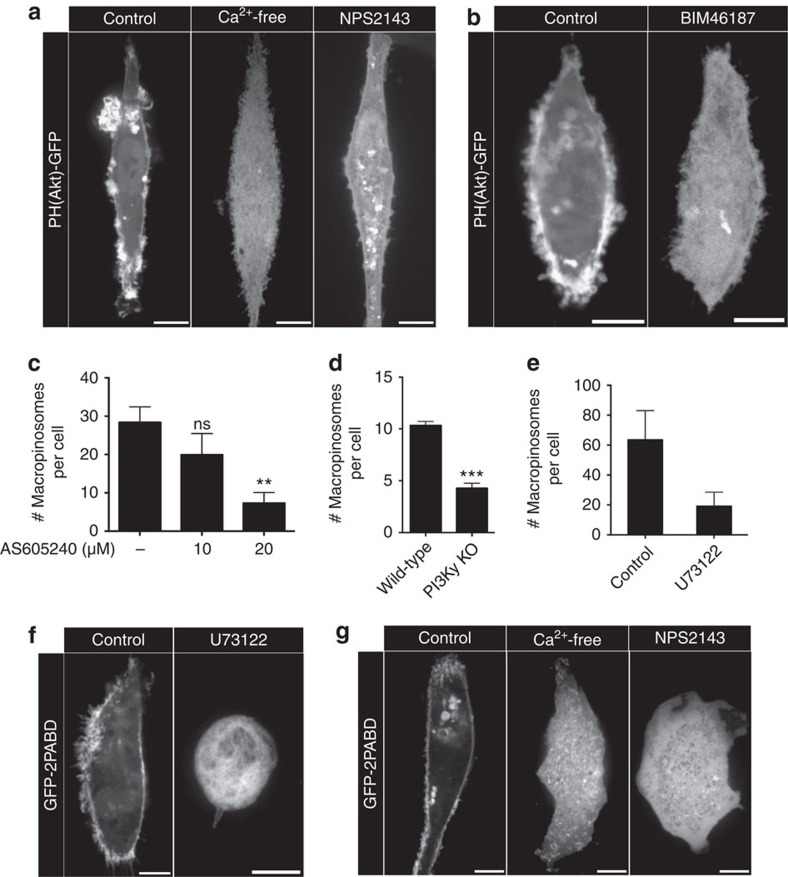
CaSR signals through a Gα-, PtdIns3Kγ- and PLC-dependent pathway. (**a**) hMDMs were transfected with the PtdIns(3,4,5)P_3_ probe (PH)Akt-GFP and imaged live in either calcium-containing or calcium-free medium. Where indicated, the cells were treated with NPS2143 (10 μM) for 1 h before imaging. (**b**) hMDMs were transfected with (PH)Akt-GFP and imaged live. Where indicated, the cells were treated with BIM46187 (10 μM) for 1 h before imaging. (**c**) hMDMs were pretreated with the PtdIns3Kγ inhibitor AS605240 at the indicated concentrations for 1 h and then incubated with fluorescent 70 kDa dextran (0.025 mg ml^−1^) for 15 min at 37 °C. The total number of macropinosomes per cell is plotted. (**d**) mBMDMs from wild-type and PtdIns3Kγ knockout mice were incubated with fluorescently labelled 70 kDa dextran (0.025 mg ml^−1^) for 15 min at 37 °C. The total number of macropinosomes per cell is plotted. (**e**) hMDMs were pretreated with the PLC inhibitor U73122 (1 μM) for 1 h and then incubated with fluorescently labelled 70 kDa dextran (0.025 mg ml^−1^) for 15 min at 37 °C. The total number of macropinosomes per cell is plotted. (**f**) hMDMs were transfected with the PtdOH probe GFP-2PABD and were treated with U73122 (1 μM) for 1 h before imaging. (**g**) hMDMs were transfected with GFP-2PABD and imaged live in either calcium-containing or calcium-free medium. Where indicated, the cells were treated with NPS2143 (10 μM) for 1 h before imaging. Scale bar, 10 μm. **P≤0.01, ***P≤0.001, NS, not significantly different.

Generation of PtdIns(3,4,5)P_3_ is required for the completion of macropinocytosis^7^,^35^,^36^. We explored whether PtdIns3K is involved in the activation of constitutive macropinocytosis by CaSR. It is noteworthy that the Gα subunit of heterotrimeric G proteins, which was shown above to be engaged by CaSR, can activate PtdIns3Kγ (refs 37, 38). By transfecting into hMDMs a biosensor consisting of the PH domain of Akt fused to green fluorescent protein (GFP) (PH(Akt)-GFP), we detected the presence of PtdIns(3,4,5)P_3_ at the plasma membrane, with noticeable enrichment at sites of ruffling (Fig. 6a; Supplementary Movie 4). Strikingly, removal of extracellular calcium or pharmacological inhibition of CaSR resulted in depletion of PtdIns(3,4,5)P_3_ from the membrane and loss of membrane ruffles (Fig. 6a; Supplementary Movie 5). The accumulation of PtdIns(3,4,5)P_3_ in ruffles required Gα-dependent signalling, as BIM46187 caused dissociation of PH(Akt)-GFP from the plasma membrane and loss of ruffles (Fig. 6b). These data suggest that tonic stimulation of CaSR is required to maintain PtdIns3K activity in resting hMDMs. To determine whether PtdIns3Kγ was responsible for the generation of PtdIns(3,4,5)P_3_ and the macropinocytosis induced by CaSR, we treated hMDMs with AS605240, a PtdIns3Kγ-specific inhibitor. AS605240 caused a dose-dependent decrease in constitutive macropinocytosis (Fig. 6c). We also generated mBMDMs from PtdIns3Kγ knockout mice and compared the efficiency of macropinocytosis to mBMDMs isolated from wild-type mice. Constitutive macropinocytosis was markedly reduced in mBMDMs lacking PtdIns3Kγ (Fig. 6d).

The generation of PtdIns(3,4,5)P_3_ fosters the recruitment of PLCγ through its PH domain. PLCγ, along with PtdIns3K activity, serves to deplete PtdIns(4,5)P_2_ from sites of macropinocytosis, allowing for the release of PtdIns(4,5)P_2_-binding proteins and actin remodelling^39^. Indeed, blocking PLC with U73122 causes inhibition of growth factor-induced macropinocytosis in epithelial cells^40^. Inhibition of PLC also inhibited constitutive macropinocytosis in hMDMs (Fig. 6e). PLC catalyses the hydrolysis of PtdIns(4,5)P_2_, releasing DAG that can be converted by diacylglycerol kinases into PtdOH; the latter is required for constitutive macropinocytosis in dendritic cells^8^. Using a biosensor consisting of the Spo20p domain fused to GFP (GFP-2PABD)^8^, we confirmed the presence of PtdOH at the plasma membrane and in membrane ruffles of hMDMs (Fig. 6f). The generation of DAG by PLC is required for PtdOH accumulation, since U73122 treatment resulted in detachment of the GFP-2PABD probe from the membrane (Fig. 6f). Importantly, inhibition of CaSR signalling by removal of extracellular calcium or by addition of NPS2143 also resulted in loss of PtdOH from the membrane (Fig. 6g). These data support a pathway in which the Gα-dependent activation of PtdIns3Kγ upon CaSR stimulation results in the generation of PtdIns(3,4,5)P_3_ at the plasma membrane, which in turn recruits PLCγ, allowing for the hydrolysis of PtdIns(4,5)P_2_ and the generation of DAG and PtdOH that facilitate constitutive macropinocytosis^8^.

### Inhibition of CaSR impairs constitutive ruffling

**Figure 7:**
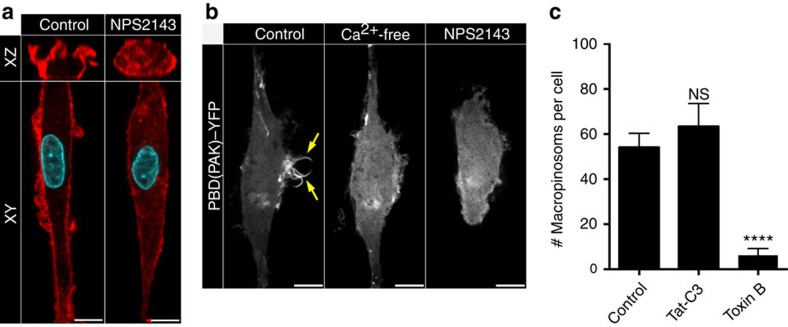
Inhibition of CaSR results in cytoskeletal changes in MDMs. (**a**) hMDMs were treated with NPS2143 (10 μM) and then fixed and labelled with fluorescently labelled phalloidin (red) and DAPI (cyan). (**b**) hMDMs were transfected with the active Rac1/Cdc42 biosensor PBD(Pak)-YFP and imaged live in either calcium-containing or calcium-free medium. Where indicated, the cells were treated with NPS2143 (10 μM) before imaging. Scale bar, 10 μm. (**c**) hMDMs were pretreated with either the *C. difficile* toxin B (50 ng ml^−1^) or Tat-C3 (10 μg ml^−1^; see Methods) and then incubated with labelled 70 kDa dextran (0.025 mg ml^−1^) for 15 min at 37 °C. The total number of macropinosomes per cell was then plotted. ****P≤0.0001, NS, not significantly different.

PtdIns(3,4,5)P_3_ and PtdOH at the membrane serve to recruit GEFs that activate Rho-family GTPases such as Rac1 (ref. 8); this in turn can initiate membrane ruffling and macropinocytosis. To establish whether CaSR utilizes this pathway we compared the actin cytoskeleton and assessed the activation of GTPases in control and NPS2143-treated hMDMs. Phalloidin staining revealed clearly defined cortical F-actin and F-actin-rich membrane ruffles extending from the surface of untreated hMDMs (Fig. 7a). In NPS2143-treated cells the band of cortical F-actin was thinner, abnormal F-actin puncta appeared in the cytoplasm, and virtually no actin-containing ruffles were observed (Fig. 7a). Differential interference contrast microscopy of live cells confirmed that dynamic membrane ruffling was arrested by NPS2143 (Supplementary Movies 2 and 3).

Rac1 and Cdc42 are thought to drive the actin remodelling required for macropinocytosis^17^. To monitor the activation state of these GTPases we employed a construct encoding PAK(PBD), a biosensor for GTP-bound Rac1 and Cdc42. hMDMs electroporated with PAK(PBD) conjugated to YFP were imaged under control and calcium-free conditions, as well as immediately after treatment with NPS2143. As expected, PAK(PBD) accumulated robustly and in a highly localized fashion in the membranes subtending macropinocytic ruffles in control cells (Fig. 7b; Supplementary Movie 6). In sharp contrast, the biosensor dissociated from the plasmalemma and became exclusively cytosolic when calcium was removed or when CaSR was inhibited (Fig. 7b; Supplementary Movie 7). These results strongly suggest that CaSR-driven signals promote activation of Rac1 and/or Cdc42. Consistent with this, we found that macropinosome formation by hMDMs was profoundly depressed by *C. difficile* toxin B which is known to inhibit Rac1 and Cdc42 (as well as other members of the Rho-GTPase family)^41^, but not by the C3 toxin of *Clostridium botulinum* that specifically inhibits RhoA (Fig. 7c). These data imply that CaSR-dependent activation of Rac and/or Cdc42 is implicated in constitutive membrane ruffling and macropinocytosis in hMDMs.

### Macropinocytosis facilitates the uptake of NOD2 ligands

**Figure 8:**
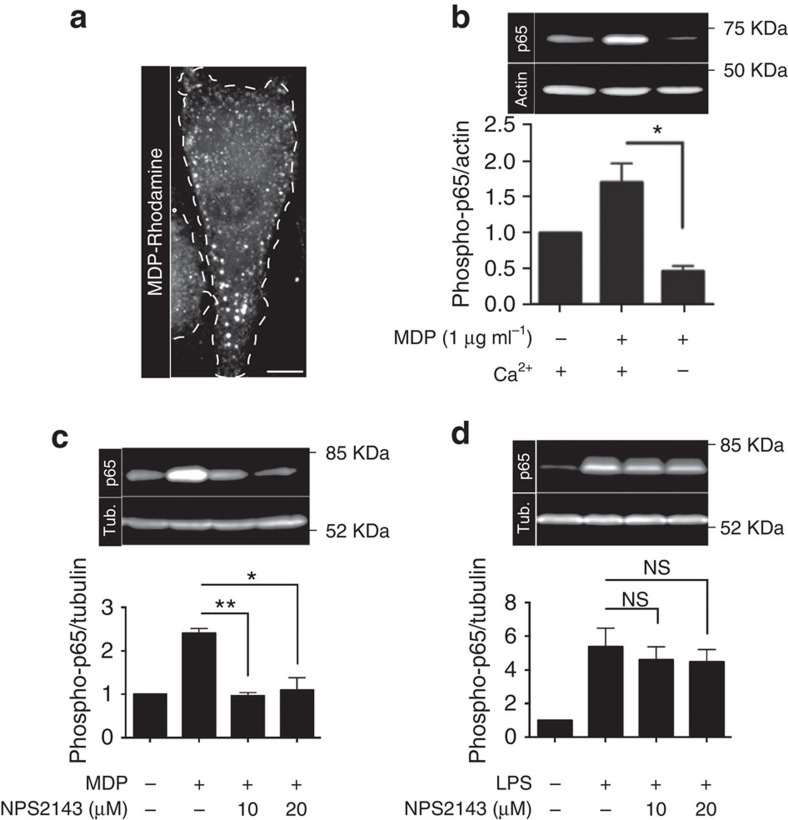
CaSR-dependent macropinocytosis delivers NOD2 ligands to the cytosol. (**a**) hMDMs were incubated with the fluorescently labelled NOD2 ligand muramyl dipeptide (MDP-rhodamine; 1 μg ml^−1^) for 15 min at 37 °C. Cells were then washed and imaged immediately by spinning disc confocal microscopy. Scale bar, 10 μm. (**b**) hMDMs were incubated in calcium-containing or calcium-free medium in the presence or absence of MDP (1 μg ml^−1^) for 15 min and then lysed, separated by 12% SDS–PAGE and subjected to immunoblotting. Image shows representative immunoblot for phosphorylated p65 (p65) and actin (used as loading control); quantification of the normalized phospho-p65/actin ratio from three independent experiments is shown below. (**c**) hMDMs were incubated with NPS2143 at the indicated concentrations in either the presence or absence of MDP (1 μg ml^−1^) for 30 min and then lysed, separated by 12% SDS–PAGE and subjected to immunoblotting. Image shows representative immunoblot for phosphorylated p65 (p65) and α-tubulin (tub.; used as loading control); quantification of the phospho-p65/α-tubulin ratio from three independent experiments is shown below. (**d**) hMDMs were incubated with NPS2143 at the indicated concentrations in either the presence or absence of LPS (0.5 μg ml^−1^) for 30 min and then lysed, separated by 12% SDS–PAGE and subjected to immunoblotting. Image shows representative immunoblot for phosphorylated p65 (p65) and α-tubulin; quantification of the phospho-p65/α-tubulin ratio from three independent experiments is shown below. *P≤0.05, **P≤0.01, NS, not significantly different.

Extracellular danger signals sensed by pattern-recognition receptors such as nucleotide-binding oligomerization domain-containing protein 1 (NOD1) and 2 (NOD2) initially enter the endocytic pathway before reaching the cytosolic compartment^42^. NOD ligands are transported in a pH-dependent manner across the membrane of endosomes/lysosomes by members of the solute carrier family of peptide transporters—SLC15A3 and SLC15A4 (ref. 42). Because large volumes of extracellular fluid are internalized in macrophages and DCs via macropinocytosis, we analysed the possible role of CaSR and of constitutive macropinocytosis in the uptake of ligands that activate pattern-recognition receptors. Fifteen minutes after being added to the medium bathing hMDMs, the fluorescently labelled NOD2 ligand muramyl dipeptide (MDP) was taken up into endomembrane vesicles; MDP was also detectable in the cytosol (Fig. 8a), presumably as a result of transport across endomembranes by peptide transporters^42^. That the ligand was being effectively sensed by NODs was indicated by the robust phosphorylation of the p65 subunit of NFκB induced by MDP (Fig. 8b,c). Strikingly, inhibition of CaSR by the removal of external calcium or with NPS2143 drastically reduced the phosphorylation of p65 elicited by MDP (Fig. 8b,c). This suggested that inhibition of CaSR-dependent constitutive macropinocytosis reduces the amount of MDP taken up and subsequently translocated to the cytosol for sensing.

To ensure that the reduction in p65 phosphorylation caused by NPS2143 was due to impaired macropinocytosis, as opposed to non-specific effects of the drug on the NFκB signalling cascade, we incubated hMDMs with lipopolysaccharide (LPS) that stimulates NFκB through Toll-like receptor 4 (TLR4). TLR4 senses LPS at the cell surface and does not require internalization. As shown in Fig. 8d, LPS (0.5 μg ml^−1^) induced robust phosphorylation of p65. Importantly, treatment with NPS2143 had no discernible effect on LPS-induced p65 phosphorylation (Fig. 8d). Comparable results were obtained using lower concentrations of LPS (10 ng ml^−1^; Supplementary Fig. 6). To ensure that the stimulation was not caused by LPS internalization due to macropinocytosis, we also performed similar experiments in cells treated with either latrunculin A or NPS2143. The inhibition of CaSR-dependent macropinocytosis or of macropinocytosis in general had no significant effect on LPS-induced phosphorylation of p65 (Supplementary Fig. 6). Altogether, these data demonstrate that constitutive macropinocytosis, which is initiated and maintained by CaSR, aids in the sentinel function of phagocytes by facilitating the uptake of pattern-recognition receptor ligands.

## Discussion

Unlike most cell types, DCs and macrophages display constitutive macropinocytosis, a unique behaviour that appears well suited for their sentinel function. This distinguishing feature has been proposed to serve as a mechanism for the acquisition of antigen for presentation on class I and II major histocompatibility molecules^13^,^20^,^43^. Indeed, inhibition of macropinocytosis markedly reduces the efficiency of antigen presentation by DCs both *in vitro* and *in vivo*^44^. The signals that initiate and maintain this constitutive process, however, have remained largely unknown. Here we show that extracellular calcium is a key stimulant of the signalling pathways that trigger constitutive macropinocytosis in macrophages and DCs. Further, we identify CaSR as the receptor that senses extracellular calcium and initiates the aforementioned signalling pathways.

CaSR is ubiquitous, but is expressed at particularly high levels in the parathyroid gland and kidney^45^,^46^, where it plays a critical role in extracellular calcium homeostasis. In myeloid osteoclast precursors CaSR directs migration towards bones, allowing for the initiation of bone remodelling^47^. CaSR, however, is also expressed in a number of tissues that are seemingly not involved in calcium homeostasis^45^, including monocytes, macrophages and DCs^28^,^48^, where it has been speculated to regulate immune function by controlling the activation of the inflammasome^28^. Our findings extend the immune function of CaSR in myeloid cells. It is relevant that extracellular calcium is elevated at sites of inflammation and injury^49^. CaSR may function in the migration towards, and acquisition of antigen at sites of injury and inflammation, thereby assisting the immune surveillance function of antigen-presenting cells.

Although previous work identified various signalling intermediates required for constitutive macropinocytosis, such as PtdIns3K, PLC and Rac1, efforts to identify the initiating event had failed^8^. We show here that CaSR can trigger the pathways that underlie constitutive macropinocytosis. We demonstrate that removal of extracellular calcium or pharmacological inhibition of CaSR block the steady state formation of PtdIns(3,4,5)P_3_ as well as the constitutive activation of Rac1/Cdc42 (Figs 6 and 7). The activation of PtdIns3K facilitates the recruitment of PLC, which, in turn, catalyses the formation of DAG at the plasma membrane. DAG not only activates protein kinase C, but can be converted to PtdOH by diacylglycerol kinases. The generation of PtdOH—which can recruit Rac GEFs bearing a polybasic domain, such as TIAM1—is a requirement for constitutive macropinocytosis^8^. In accordance with this model, inhibition of CaSR depletes PtdOH from the plasma membrane (Fig. 6g). We believe that the impaired activity of Rac1/Cdc42 on inhibition of CaSR (Fig. 7b) is due to diminished recruitment of PtdOH-responsive GEFs. This proposal is consistent with observations we made in hMDMs transfected with a TIAM1-GFP construct: in otherwise untreated cells TIAM1-GFP localized to the plasma membrane, where it induced pronounced membrane ruffling (Supplementary Movie 8). Interestingly, the machinery for constitutive macropinocytosis seems to be present in most cells, as the heterologous expression of CaSR in HEK cells confers the ability to perform macropinocytosis to these otherwise quiescent cells (Fig. 4).

Calcium had also been reported to facilitate macropinocytosis during growth cone collapse in neurons (4). In this case, however, it appears that changes in cytosolic calcium—as opposed to stimulation of CaSR by exofacial calcium—are responsible for the observed effect, which was promoted by release of ryanodine-sensitive (endoplasmic reticulum) calcium stores. Whether extracellular calcium also contributes to macropinocytosis in this system or in transformed cells where Ras is mutated remains to be defined.

In addition to identifying its initiating factor, the present work provides some insight into the functional relevance of constitutive macropinocytosis. Unlike growth factor-induced macropinocytosis, which is coupled to an increased metabolic demand for cell growth and proliferation, constitutive macropinocytosis does not appear to support increased cellular metabolism and is likely to serve a very different function. To our knowledge, constitutive macropinocytosis is a unique feature of innate immune cells and, as discussed earlier, is coupled to the uptake of antigen^13^,^20^,^43^,^44^,^50^. Bulk fluid uptake, however, is not a particularly efficient mechanism for antigen internalization. In line with this, binding of antigens to specific receptors on B cells—immunoglobulins—increases the efficiency of their internalization and ultimate presentation to T cells ∼1,000-fold^51^. DCs and macrophages also express receptors to enhance the uptake of antigens, such as Fc and scavenger receptors.

We also showed a novel function for the constitutive macropinocytosis of innate immune cells. Specifically, constitutive macropinocytosis serves as a major conduit for the delivery of ligands sensed by pattern-recognition receptors. Indeed, inhibition of constitutive macropinocytosis—attained by blocking CaSR—drastically reduced the ability of hMDMs to sense the NOD2 ligand, MDP (Fig. 8). By analogy, we predict that macropinocytosis serves also to deliver ligands to TLRs located in endomembrane compartments, such as TLR7 and TLR9.

In summary, a major distinguishing feature between growth factor-induced and constitutive macropinocytosis is the requirement for extracellular calcium. Extracellular calcium is sensed by the G-protein-coupled receptor CaSR, which initiates a signalling cascade that allows for the constitutive extension of membrane ruffles and the formation of macropinosomes. These structures not only facilitate the uptake of antigens, but they also serve as a major route of uptake and delivery to the cytosol of pattern-recognition receptor ligands.

## Methods

### Reagents

Tetramethyl rhodamine-conjugated 70,000 MW dextran, Alexa Fluor 488 conjugated transferrin, EGF, Superscript VILO cDNA synthesis kit, and the anti-GFP antibody (catalogue number, A11120; dilution, 1:1,000) and Fura2-AM were acquired from Life Technologies (Carlsbad, CA, USA). 5-(*N*-ethyl-*N*-isopropyl)amiloride, LY294002, thapsigargin, NPS2143, Calhex 231, PTX, LPS, DAPI, latrunculin A, the anti-actin monoclonal antibody (catalogue number, A5441; dilution, 1:1,000) and the anti-tubulin monoclonal antibody (catalogue number, T5168; dilution, 1:1,000) were purchased from Sigma-Aldrich (St Louis, MO, USA). Human and murine recombinant M-CSF, IL-4 and GM-CSF were from Peprotech (Rocky Hill, NJ, USA). AS605240 was from Selleck Chemicals (Houston, TX, USA). EGTA and EDTA were from BioShop Canada (Burlington, ON, Canada). The phospho-VASP (catalogue number, 3111 S; dilution, 1:500) and phospho-p65 (catalogue number, 3033 S; dilution, 1:500) antibodies were from Cell Signaling Technology (Beverley, MA, USA). Forskolin and U73122 were from Tocris Bioscience (Bristol, UK). MDP and MDP-rhodamine were from InvivoGen (San Diego, CA, USA). *C. difficile* toxin B was purchased from TechLab (Blacksburg, VA, USA). Tat-C3 was from Cytoskeleton Inc. (Denver, CO, USA). Ionomycin was from EMD (Billerica, MA, USA). HOE-694 was the gift of Dr H.J. Lang (Aventis Pharma, Frankfurt am Main, Germany). The SeP-CaSR construct was provided by Dr Jeremy Henley (University of Bristol, Bristol, UK). BIM46187 was synthesized as described in ref. 29. The forward (5′-CAGGTATAATTTCCGTGGGT-3′) and reverse (5′-GTACTGGGAGATGAGTTCAC-3′) primers for the CaSR RT–PCR and the forward (5′-TTCCAATATGATTCCACCCA-3′) and reverse (5′- CATACCAGGAAATGAGCTTG-3′) primers for the GAPDH RT–PCR were purchased from Integrated DNA Technologies (Coralville, IA, USA). The GeneJET RNA purification kit was from Thermo Fisher Scientific (Waltham, MA, USA). The TaqMan gene expression mastermix and the CaSR TaqMan gene expression assay were from Applied Biosystems (Waltham, MA, USA).

### Solutions

The calcium-free medium contained 1 mM KH_2_PO_4_, 154 mM NaCl, 5.6 mM Na_2_HPO_4_, 2 mM MgCl_2_, 2 mM EGTA and 10 mM glucose, pH 7.2 at 37 °C. The calcium-containing medium contained 1 mM KH_2_PO_4_, 154 mM NaCl, 5.6 mM Na_2_HPO_4_, 2 mM MgCl_2_, 2 mM CaCl_2_ and 10 mM glucose, pH 7.2 at 37 °C. The Na^+^-rich buffer contained 140 mM NaCl, 3 mM KCl, 1 mM MgCl_2_, 5 mM glucose, and 20 mM 4-(2-hydroxyethyl)-1-piperazineethanesulfonic acid (HEPES) adjusted to pH 7.2 at 37 °C.

### Macrophage and dendritic cell isolation and cell culture

For hMDMs and hMDDCs, peripheral blood mononuclear cells were isolated from the blood of healthy donors by density-gradient separation with Lympholyte-H (Cedarlane, Burlington, ON, Canada). Monocytes were separated from other mononuclear cells in the washed buffy coat by adherence to glass coverslips in 12-well plates at a density of 3.0 × 10^6^ mononuclear cells per well. Monocytes were then cultured for 7 days in RPMI 1640 supplemented with 10% heat-inactivated fetal bovine serum, antibiotic/antimycotic (MultiCell, Wisent, St Bruno, Canada) and either 25 ng ml^−1^ GM-CSF+20 ng ml^−1^ IL-4 (for hMDDCs) or 25 ng ml^−1^ M-CSF (for hMDMs). The medium and cytokines on the cells were aspirated and replaced every 2–3 days.

For murine BMDMs, bone marrow was collected from the long bones of healthy wild-type or PtdIns3Kγ knockout mice. The PtdIns3Kγ knockout mice were generated^52^ and kindly provided by Dr J. M. Penninger (Institute of Molecular Biotechnology of the Austrian Academy of Sciences, Vienna, Austria). The precursor cells (plated at a density of 4.5 × 10^5^ cells per 10-cm dish) were cultured in the presence of 10 ng ml^−1^ M-CSF in RPMI 1640 supplemented with 10% heat-inactivated fetal bovine serum and antibiotic/antimycotic for 7 days. The medium and cytokines on the plate were aspirated and replaced every 2–3 days.

A431 and HEK293 cells were obtained from the American Type Culture Collection (ATCC, Rockville, MD, USA). Cells were maintained in DMEM (MultiCell, Wisent, St Bruno, Canada) supplemented with 10% heat-inactivated fetal bovine serum. When cultures reached confluence, they were detached using 0.05% trypsin-EDTA and replated on either coverslips for macropinocytosis assays or in flasks for maintenance.

### Transfections

For hMDMs, DNA transfections were performed using the Neon transfection system (Life Technologies, Carlsbad, CA, USA). hMDMs were suspended using Accutase (Gibco, Life Technologies, Carlsbad, CA, USA) and the pellet was washed 3 ×. Transfections were performed according to the manufacturer's protocol. For siRNA transfections of hMDMs, the HiPerFect transfection reagent (Qiagen, Hilden, Germany) was used according to the manufacturer's protocol. For HEK cells, transfections were performed with the FuGENE 6 transfection reagent (Promega, Madison, WI, USA) according to the manufacturer's instructions.

### PCR

To detect the expression of CaSR messenger RNA (mRNA) in hMDMs and HEK cells, one step RT–PCR was performed. RNA was purified from hMDMs and HEK cells using the GeneJet RNA purification kit (Thermo Fisher Scientific) and used as a template for the generation of complementary DNA (cDNA) and subsequent PCR amplification using the OneStep RT–PCR kit from Life Sciences according to the manufacturer's instructions. The primary sequences of the forward and reverse primers are listed in the ‘Reagents' section. The amplification products were visualized by electrophoresis on agarose gels prestained with ethidium bromide. Uncropped gels are presented in Supplementary Fig. 7a.

To quantify the expression of CaSR mRNA after siRNA knockdown, RNA was purified from hMDMs using the GeneJet RNA purification kit (Thermo Fisher Scientific) and used as a template for the generation of cDNA using the Superscript VILO cDNA synthesis kit (Life Technologies). The CaSR-specific Taqman gene expression assay (Life Technologies) was then used for real-time quantitative PCR to quantify the expression of CaSR, which was normalized relative to actin mRNA.

### Macropinocytosis assay

Cells were plated on 18 mm coverslips 24 h before performing the macropinocytosis assay. In the case of drug treatments, cells were pretreated with their respective drugs for 1 h before the assay, except for PTX, Tat-C3 and *C. difficile* toxin B, which required 24, 4 and 4 h pretreatments, respectively. The concentrations for the drugs used are as follows: EIPA (10 μM), HOE-694 (10 μM), latrunculin A (2 μM), LY294002 (10 μM), PTX (0.1 μg ml^−1^), BIM46187 (10 μM), NPS2143 (1, 5, 10 or 20 μM), forskolin (10 μM), U73122 (1 μM), *C. difficile* toxin B (50 ng ml^−1^), Tat-C3 (10 μg ml^−1^) and AS605249 (5, 10 or 20 μM). After pretreatment, the medium was aspirated from the cells and replaced with either calcium-containing or calcium-free medium with rhodamine-conjugated 70 kDa dextran (25 μg ml^−1^) and either M-CSF (200 ng ml^−1^) or EGF (200 ng ml^−1^) where indicated. Cells were incubated at 37 °C for 15 min and then washed and imaged immediately. Imaging was done by spinning disk confocal microscopy on an Axiovert 200 M equipped with a 63 × objective and a separate 1.5 × magnifying lens (Carl Zeiss, Oberkochen, Germany). The microscope was fitted with a piezo focus drive and diode-pumped solid-state lasers (440, 491, 561, 638 and 655 nm; Spectral Applied Research, Richmond Hill, ON, Canada). Micrographs were acquired using a CCD camera (Hamamatsu Photonics, Hamamatsu, Japan) under the control of Volocity software. Macropinosomes were then counted from the acquired images using the measurement tool in the ImageJ software.

### Intracellular calcium measurements

MDMs were loaded with Fura2-AM at a concentration of 3 μM in HBSS (MultiCell, Wisent, St Bruno, Canada) for 20 min at 37 °C. They were then washed with fresh HBSS 3 × and incubated at 37 °C for an additional 10 min. hMDMs were then placed into a magnetic Chamlide coverslip holder, covered in HBSS and placed onto the temperature controlled (37 °C) microscope stage. The hMDMs were imaged on a Leica DM IRB microscope (Leica, Wetzlar, Germany). Calcium measurements were done by fluorescence ratio imaging using a filtre wheel (Sutter Instruments, Novato, CA, USA) to rapidly switch between excitation filters. Fura2 was excited by light from an X-Cite 120 lamp (EXFO Photonic Solutions, Quebec, Canada) using dual excitation (340 and 380 nm) and single emission (510 nm). The emitted light was captured by a CCD camera (Cascade II; Photometrics, Tucson, AZ, USA). The filtre wheels, shutters and camera were controlled by the MetaFluor software (MDS Analytical Technologies, Sunnyvale, CA, USA).

After experimental measurements were taken, ionomycin was added at a concentration of 6 μM to acquire an *R*_max_ (340/380) value for approximation of absolute [Ca^2+^]_I_ values. The Grynkiewicz formula^53^ was used to convert the fluorescence ratios to [Ca^2+^]_I_ values.

### Immunoblotting

Cells were washed 3 × with ice-cold PBS and were subsequently lysed using a small volume of ice-cold RIPA buffer. Total protein concentration was calculated using the Bradford assay and 50 μg of protein of each sample was loaded and separated by 12% SDS–PAGE. The protein was transferred to a polyvinylidene difluoride (PVDF) membrane and blocked in Tris-buffered saline containing 0.05% Tween-20 (TBST) and 10% fat-free milk for 30 min. The primary antibody was added to the membrane in 2.5% milk in TBST for 1 h at room temperature and washed 3 × with TBST for 10 min each. The membrane was reblocked with 10% milk in TBST for 15 min and the HRP-conjugated secondary antibodies were added in 2.5% milk in TBST for 45 min. The membrane was washed 3 × with TBST for 10 min each and blot was visualized on the Odyssey Fc (LI-COR, Lincoln, NE, USA). Band intensity was quantified using the ImageStudio Lite software. Uncropped versions of all the gels presented can be seen in Supplementary Fig. 7.

### Data presentation and statistics

Unless otherwise indicated, images are representative of (≥50) cells from at least three separate experiments. Data presented in text and graphs are the means plus standard error of at least three independent experiments. Unpaired *t*-tests were used to establish the significance of experimentally observed differences.

## Additional information

**How to cite this article:** Canton, J. *et al*. Calcium-sensing receptors signal constitutive macropinocytosis and facilitate the uptake of NOD2 ligands in macrophages. *Nat. Commun.* 7:11284 doi: 10.1038/ncomms11284 (2016).

## Supplementary Material

Supplementary Figures Supplementary Figures 1-7

Supplementary Movie 1 Persistence of macropinosomes and limited mixing with late endosomal/lysosomal contents during the first 15 minutes after uptake 70 kDa dextran. hMDMs were pulsed with Alexa 647-labeled 10 kDa dextran (0.025 mg/mL) for 1 hour followed by a chase at 37°C to label the late endosomal/lysosomal compartment (cyan, upper panel). The hMDMs were then incubated with rhodamine-labeled 70 kDa dextran (0.025 mg/mL) for 1 min at 37°C, in the presence of M-CSF (200 ng/mL) to label newly formed macropinosomes (red, middle panel). The cells were then washed and imaged on a heated stage acquiring an image every 30 seconds for 15 min. The video is displayed at 7 frames per sec. Scale bar = 10 μm.

Supplementary Movie 2 hMDMs extend membrane ruffles constitutively. hMDMs were imaged by differential interference contrast microscopy in calcium-containing medium for 15 min, acquiring images every 30 sec. The video is displayed at 7 frames per sec.

Supplementary Movie 3 Pharmacological inhibition of CaSR arrests constitutive membrane ruffling. hMDMs were pretreated with NPS2143 (10 μM) and then imaged in calcium-containing medium by differential interference contrast microscopy for 15 min, acquiring images every 30 sec. The video is displayed at 7 frames per sec.

Supplementary Movie 4 PtdIns(3,4,5)P3 is present constitutively on the plasma membrane and in the ruffles of resting MDMs. hMDMs were transfected with the PtdIns(3,4,5)P3 probe (PH)Akt-GFP and imaged live by spinning disc confocal microscopy in calcium-containing medium, acquiring images every 15 sec for 10 min. The video is displayed at 7 frames per sec.

Supplementary Movie 5 PtdIns(3,4,5)P3 levels are reduced at the plasma membrane and in the membrane ruffles upon CaSR inhibition. hMDMs were transfected with the PtdIns(3,4,5)P3 probe (PH)Akt-GFP and imaged live by spinning disc confocal microscopy in calcium-containing medium, acquiring images every 15 sec for 10 min. NPS2143 (10 μM) was added to the cells immediately prior to initiating image acquisition. Membrane ruffles retract and the (PH)Akt-GFP probe is lost from the plasma membrane. The video is displayed at 7 frames per sec.

Supplementary Movie 6 GTP-loaded Rac1/Cdc42 are present constitutively on the plasma membrane and in the membrane ruffles of resting hMDMs. hMDMs were transfected with the active Rac1/Cdc42 biosensor PBD(Pak)-YFP and imaged live by spinning disc confocal microscopy in calcium-containing medium, acquiring images every 15 sec for 5 min. The video is displayed at 7 frames per sec.

Supplementary Movie 7 GTP-loaded Rac1/Cdc42 levels are reduced upon CaSR inhibition. hMDMs were transfected with the active Rac1/Cdc42 biosensor PBD(Pak)-YFP and imaged live by spinning disc confocal microscopy in calcium-containing medium, acquiring images every 15 sec for 5 min. NPS2143 (10 μM) was added to the cells immediately prior to initiating image acquisition. Membrane ruffles retract and the PBD(Pak)-YFP probe is lost from the plasma membrane. The video is displayed at 7 frames per sec.

Supplementary Movie 8 Overexpression of TIAM1, a Rac1 GEF, induces formation of highly dynamic membrane ruffles. hMDMs were transfected with a construct encoding a fusion protein of TIAM1 fused to GFP and imaged in calcium-containing medium by spinning disc confocal microscopy, acquiring images every 30 sec for 20.5 min. The video is displayed at 7 frames per sec.
