# Supplementary material for: Calcium-sensing receptors signal constitutive macropinocytosis and facilitate the uptake of NOD2 ligands in macrophages
Source: Nat Commun. 2016 Apr 6;7:11284. doi: 10.1038/ncomms11284 (PMC4823870; doi:10.1038/ncomms11284)
Supplement: Supplementary Figures — 1-7 [file ncomms11284-s1.pdf]

SUPPLEMENTARY INFORMATION

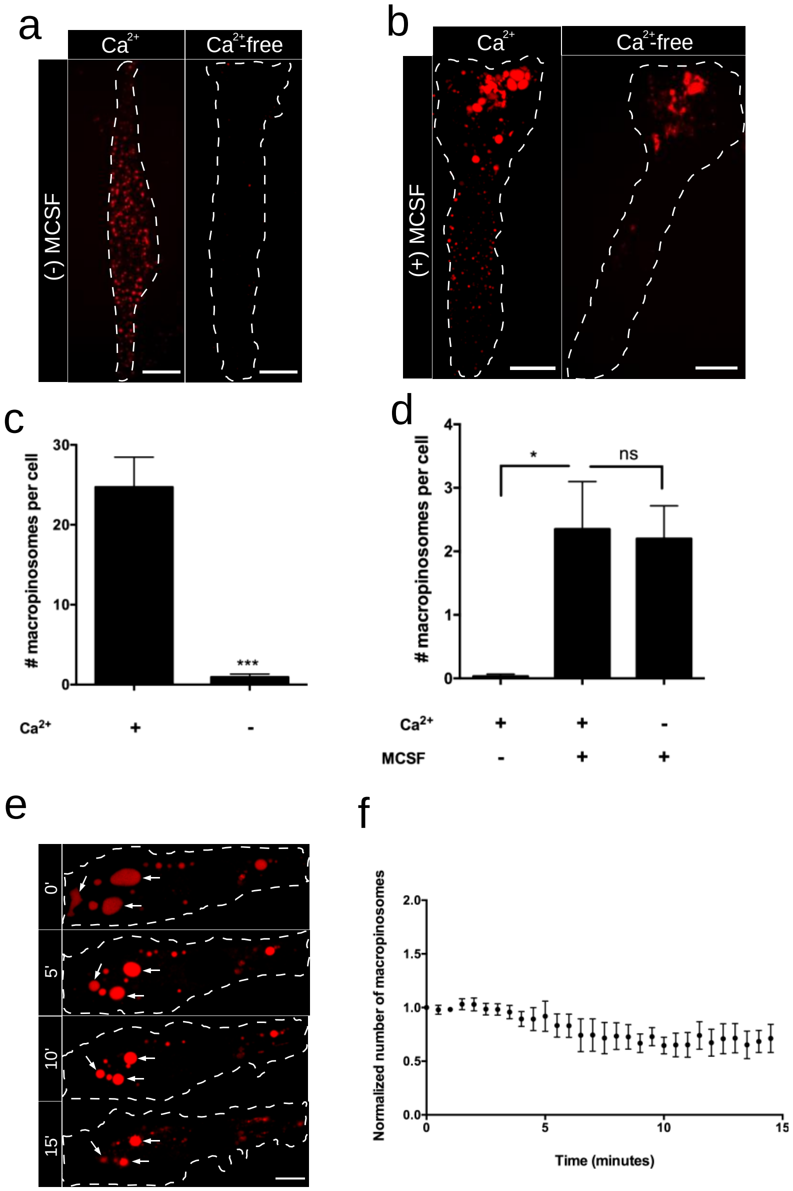

Supplementary Figure 1. Effect of external calcium removal on constitutive macropinocytosis assessed using short dextran pulses. hMDMs were incubated with fluorescently-labeled 70 kDa dextran (0.025 mg/mL) for 3 min at 37°C, in the presence **(a)** or absence **(b)** of M-CSF (200 ng/mL), in either calcium-containing or calcium-free medium (see Materials and methods). The number of macropinosomes under **(c)** or above **(d)** 3 μm in diameter is plotted. Data represent the means ± SEM of at least 3 independent experiments using cells from at least 2 separate healthy donors.

(e) hMDMs were incubated with fluorescently-labeled 70 kDa dextran (0.025 mg/mL) for 1 min at 37°C, in the presence of M-CSF (200 ng/mL), washed, and immediately imaged by confocal microscopy on a heated (37°C) stage for 15 min, acquiring an image every 30 sec. A representative cell is shown at 0, 5, 10 and 15 min after the dextran pulse. The mean number of macropinosomes per cell for 6 representative cells is plotted in **(f)** for the time points indicated. $*p \leq 0.05$, $***p \leq 0.001$, n.s. = not significantly different. Scale bars = 10 μm.

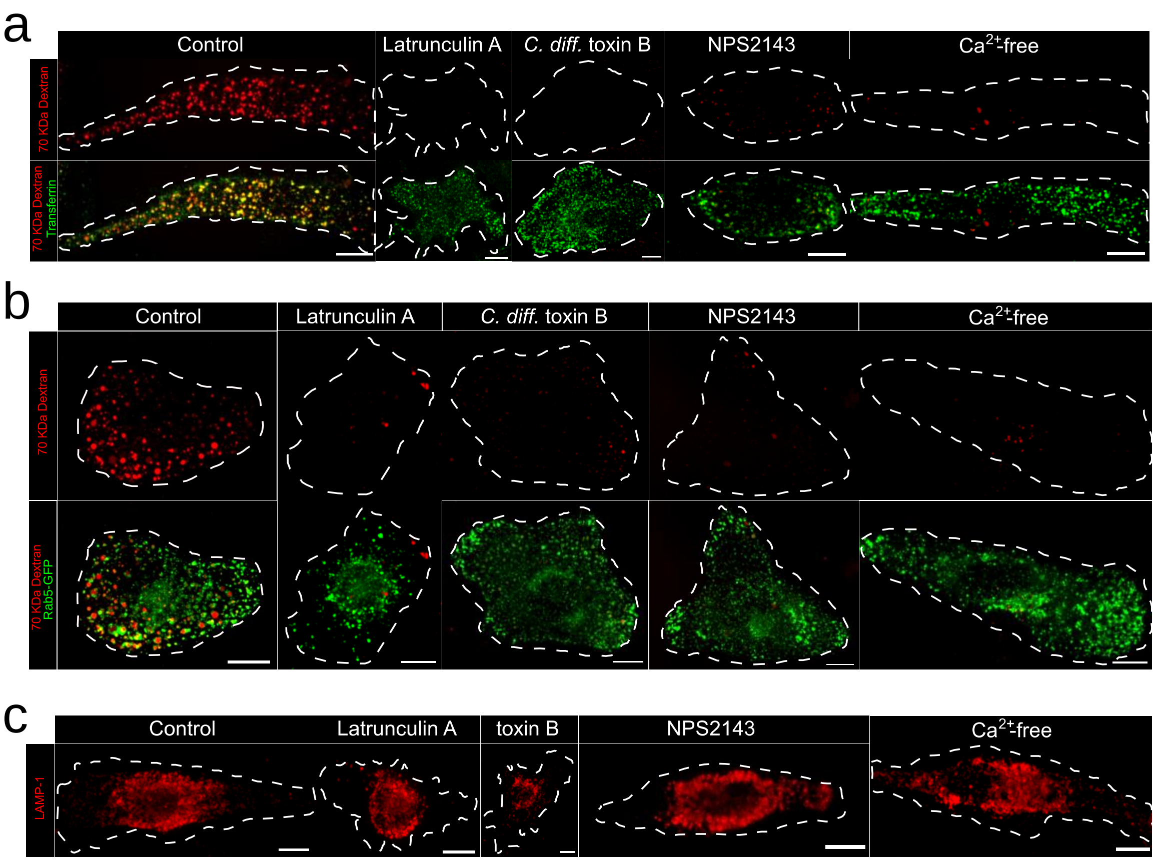

Supplementary Figure 2. External calcium removal on endocytosis. (a) hMDMs were pretreated with either latrunculin A (2 μM), *C. difficile* toxin B (50 ng/mL), NPS2143 (10 μM) or calcium-free medium. The cells were then pulsed with fluorescently-labeled 70 kDa dextran (red) and transferrin (green) for 15 min at 37°C. Cells were washed and immediately imaged by confocal microscopy. **(b)** hMDMs were transfected with Rab5-GFP and, 24 hrs after transfection subjected to the indicated treatments. The cells were then pulsed for 15 min at 37°C with labeled 70 kDa dextran (red), washed and immediately imaged by confocal microscopy. **(c)** hMDMs were treated with either latrunculin A (2 μM), *C. difficile* toxin B (50 ng/ml), NPS2143 (10 μM) or calcium-free medium as above, then fixed, permeabilized and immunostained for LAMP1 (red), and imaged by confocal microscopy. Scale bars = 10 μm.

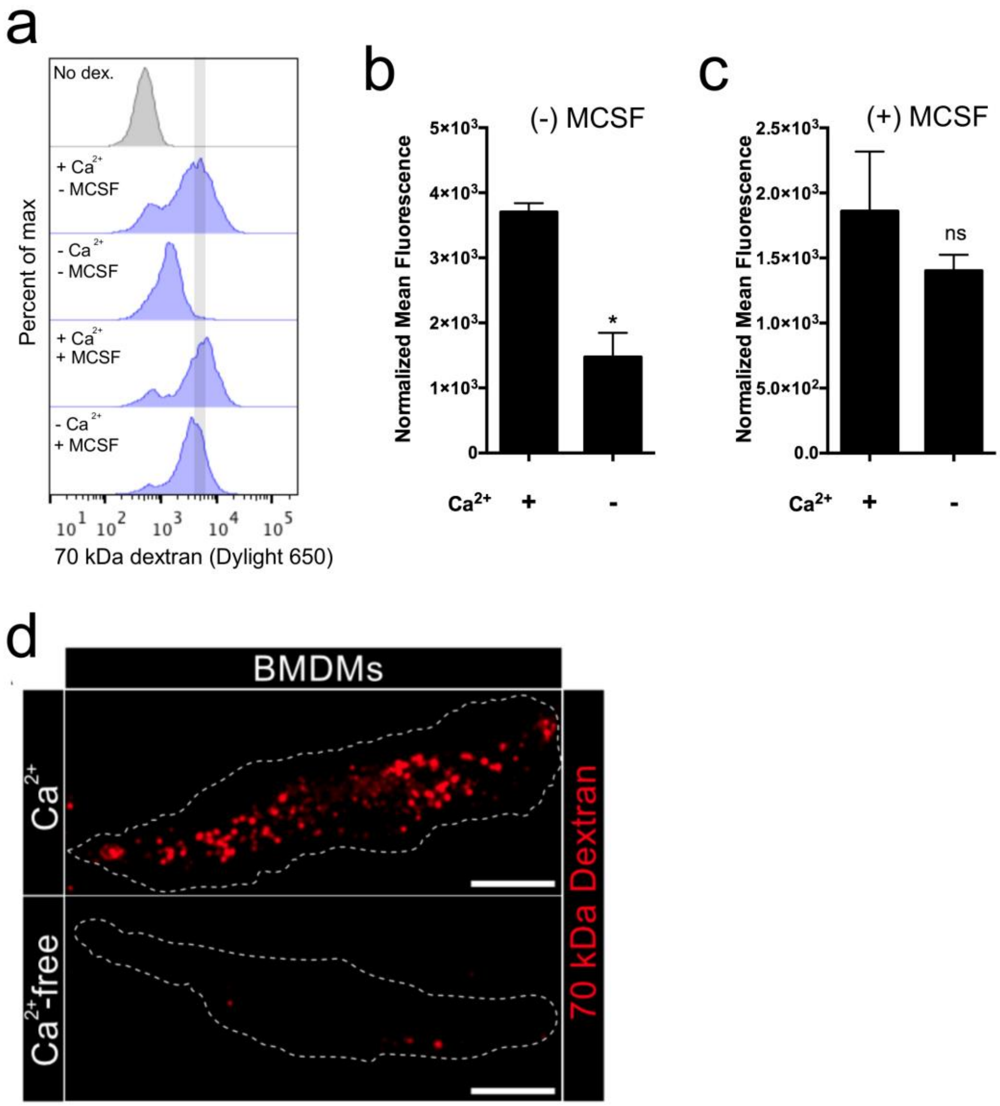

Supplementary Figure 3. Quantification of 70 kDa dextran uptake by flow cytometry and effect of calcium removal. (a) hMDMs were incubated with DyLight 650-labeled 70 kDa dextran (0.025 mg/mL) for 15 min at 37°C, in the presence or absence of M-CSF (200 ng/mL), in either calcium-containing or calcium-free medium

(see Materials and methods). The cells were then gently lifted with PBS containing 2 mM EDTA and the uptake of dextran was quantified by flow cytometry. The mean fluorescence intensity of each sample in arbitrary units is plotted in **(b)** and **(c)**. Data represent the means ± SEM of at least 3 independent experiments using cells from multiple healthy donors. Note that the uptake of dextran by cells not treated with M-CSF has been subtracted from the (+)M-CSF values, that represent the growth factor-induced component only. **(d)** Murine BMDMs were incubated with fluorescently-labeled 70 kDa dextran (0.025 mg/mL) for 15 min at 37°C in either calcium-containing or calcium-free medium and imaged by confocal microscopy. Data represent the means ± SEM of at least 3 independent experiments using cells from at least 2 separate healthy mouse donors. $^*p \leq 0.05$, n.s. = not significantly different. Scale bars = 10 μm.

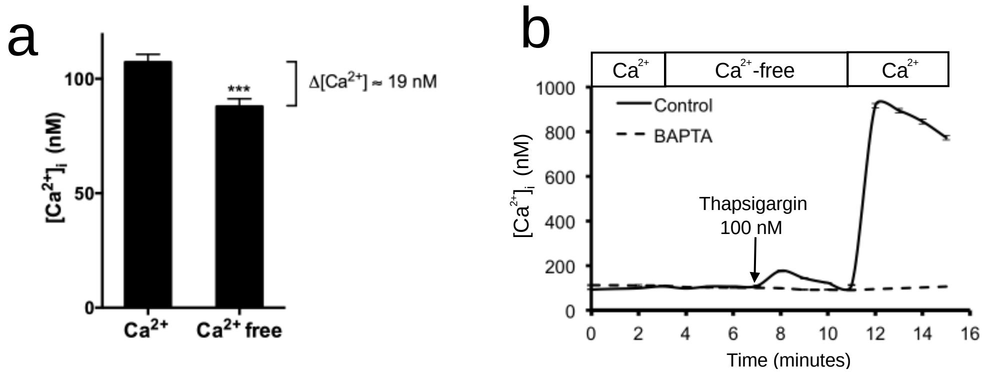

Supplementary Figure 4. Effects of external calcium removal on baseline $[Ca^{2+}]_i$. (a) hMDMs were loaded with 5 μM Fura2-AM in calcium-containing medium and then incubated in either calcium-containing or calcium-free medium for 15 min. $[Ca^{2+}]_i$ was then determined. **(b)** hMDMs were loaded with 5 μM Fura2-AM in calcium-containing medium and imaged in a magnetic Chamlide chamber at 37°C in calcium-containing medium for 3 min, followed by calcium-free medium for 8 min and then calcium-containing medium for the final 5 min. Thapsigargin (100 nM) was added where indicated; images for $[Ca^{2+}]_i$ determination were acquired every minute. ***$p \leq 0.001$.

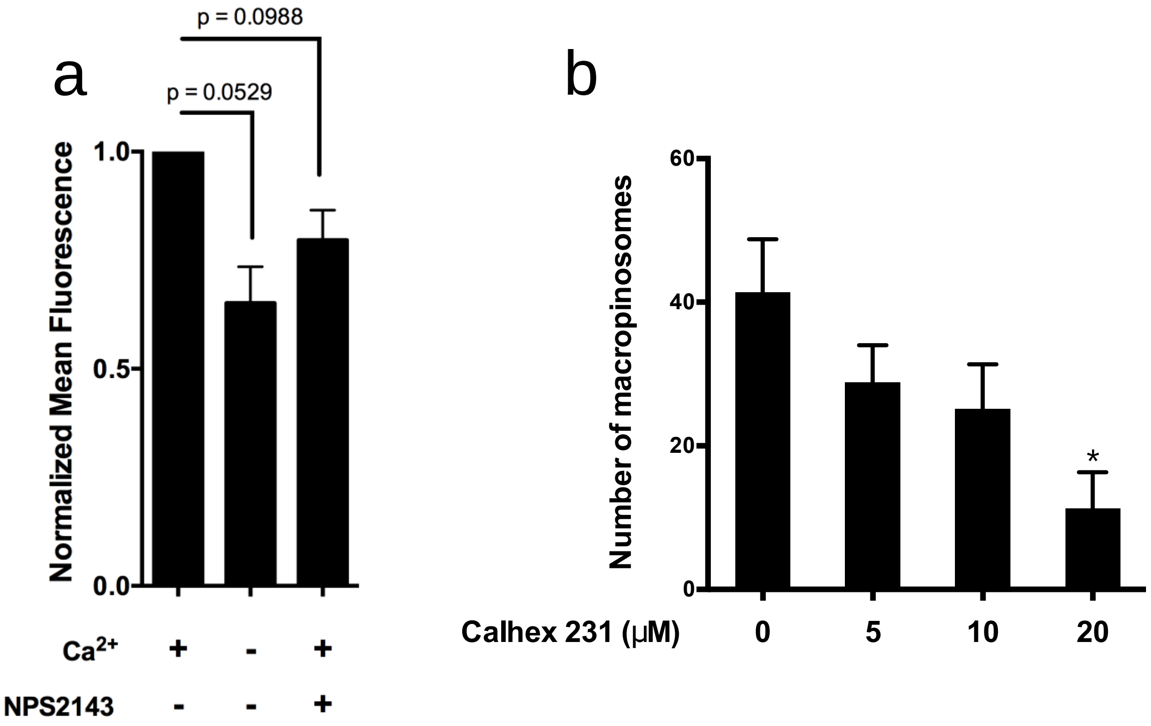

Supplementary Figure 5. Effect of CaSR antagonists on the constitutive macropinocytosis of SeP-CaSR-expressing HEK293 cells and of hMDMs. (a) Untransfected and HEK293 cells transfected with SeP-CaSR were incubated with DyLight 650-labeled 70 kDa dextran (0.025 mg/mL) for 15 min at 37°C. Cells were then gently lifted and dextran uptake assessed by flow cytometry. Gates were drawn on SeP-positive and SeP-negative populations as in figure 4**.** The effect of external calcium removal and NPS2143 treatment (10 µM for 30 min at 37°C) on dextran uptake is presented as mean fluorescence intensity normalized to control samples. Means ± SEM of 3 individual experiments. The more modest inhibition of constitutive macropinocytosis in these cells may be due to reduced accessibility of the allosteric inhibitor caused by the exofacial SeP tag (Supplementary fig. 5a). **(b)** hMDMs were pre-treated with the indicated dose of Calhex 231 and then incubated with labeled 70 kDa dextran (0.025 mg/mL) for 15 min at 37°C. The total number of macropinosomes per cell is plotted at

the indicated concentration of NPS2143. Data are means ± SEM of at least 3 independent experiments using cells from multiple healthy donors. *$p \leq 0.05$.

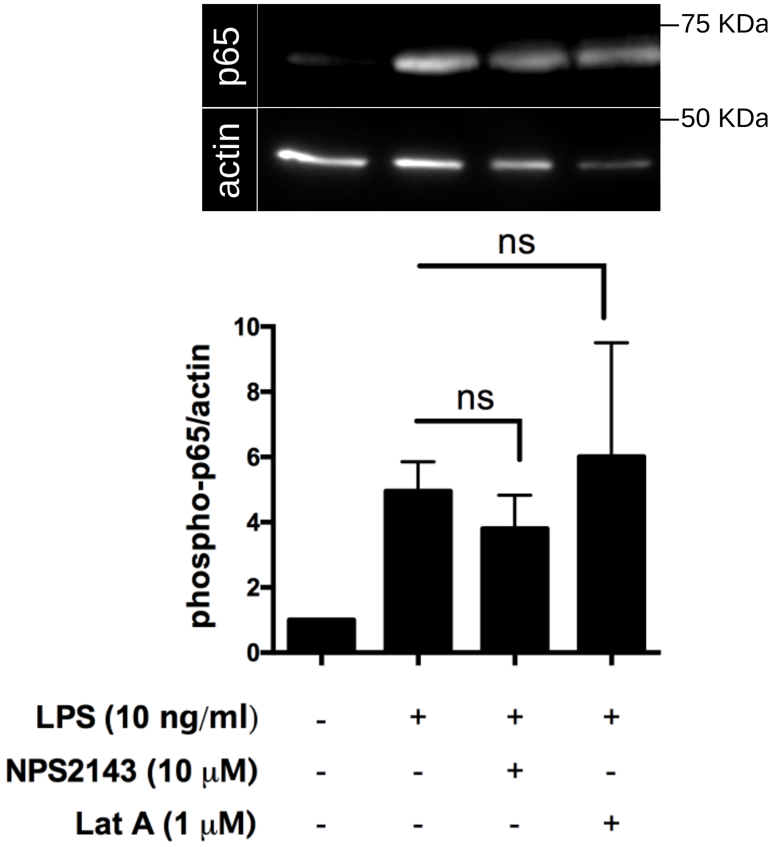

Supplementary Figure 6. Inhibiting macropinocytosis does not affect LPS signaling through NF-κB. (a) hMDMs were incubated with NPS2143 (10 μM) or latrunculin A (2 μM) in either the presence or absence of LPS (10 ng/mL) for 30 min and then lysed, separated by 12% SDS-PAGE and subjected to immunoblotting. Image shows representative immunoblot for phosphorylated p65 (p65) and actin (used as loading control); quantification of the phospho-p65/actin ratio from 3 independent experiments is shown below. n.s. = not significantly different.

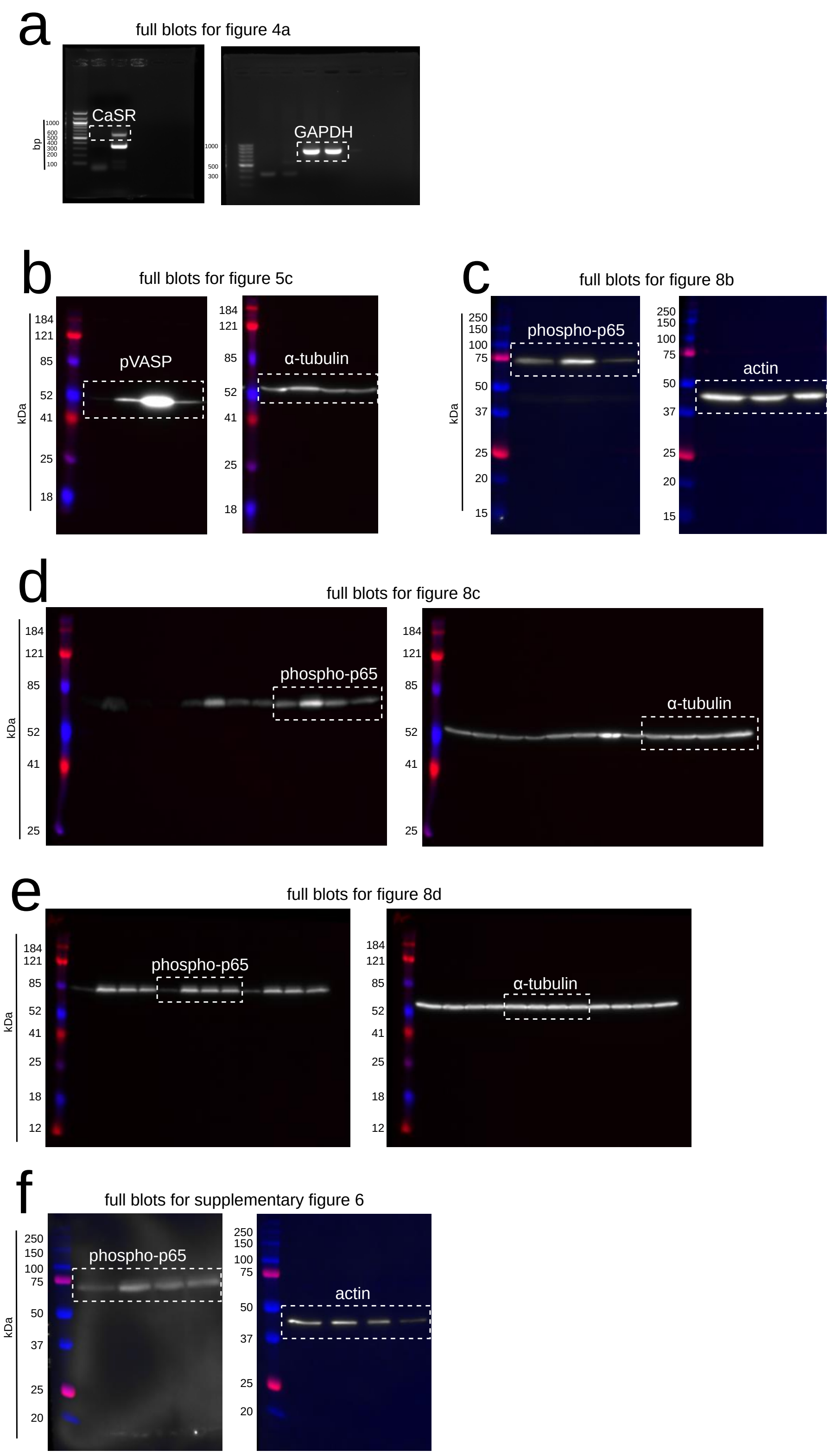

Supplementary Figure 7. Uncropped gels and blots. Images of the full agarose gels and Western blots that were cropped for display purposes are shown.

Dashed, white boxes indicate the area cropped for the main figures. **(a)** Agarose gels of the RT-PCR results shown in figure 4a. The left gel represents the PCR reaction using CaSR-specific primers and the lanes are as follows: lane 1, HEK293 cells; lane 2, hMDMs. The right gel represents the PCR reaction using CaSR-specific primers in lanes 1 and 2 and GAPDH-specific primers in lanes 3 and 4. The samples loaded into each lane are as follows: lane 1, HEK293 cells; lane 2, hMDMs; lane 3, HEK293 cells; lane 4, hMDMs. **(b)** Western blots for experiments shown in figure 5c. Samples were loaded in identical volumes for both blots and probed for either phosphorylated VASP or α-tubulin. The lanes for each blot are as follows: lane 1, untreated hMDMs; lane 2, hMDMs in Ca^{2+}-free medium; lane 3, hMDMs treated with 10 μM forskolin; lane 4, hMDMs treated with 10 μM NPS2143. **(c)** Western blots for experiments shown in figure 8b. Samples were loaded in identical volumes for both blots and probed for either phosphorylated p85 or actin. The lanes for each blot are as follows: lane1, untreated hMDMs; lane 2, hMDMs treated with 1 μg/mL MDP; lane 3, hMDMs treated with 1 μg/mL MDP in Ca^{2+}-free medium. **(d)** Western blots for experiments shown in figure 8c. Samples were loaded in identical volumes for both blots and probed for either phosphorylated p85 or α-tubulin. The lanes for each blot are as follows: lane1, untreated hMDMs; lane 2, hMDMs treated with 1 μg/mL MDP; lane 3, hMDMs treated with 1 μg/mL MDP and 10 μM NPS2143; lane 4, hMDMs treated with 1 μg/mL MDP and 20 μM NPS2143. Lanes 5-12 represent two additional repeats of the identical experiment in the same order as lanes 1-4. **(e)** Western blots for experiments shown in figure 8d. Samples were loaded in identical volumes for both blots and probed for either phosphorylated p85 or α-tubulin. The lanes for each blot are as follows: lane1, untreated hMDMs; lane 2, hMDMs treated with 0.5 μg/mL LPS; lane 3, hMDMs treated with 0.5 μg/mL LPS and 10 μM NPS2143; lane 4, hMDMs treated with 0.5 μg/mL LPS and 20 μM NPS2143. Lanes 5-12 represent two additional repeats of the identical experiment in

the same order as lanes 1-4. **(f)** Western blots for experiments shown in supplementary figure 6. Samples were loaded in identical volumes for both blots and probed for either phosphorylated p85 or actin. The lanes for each blot are as follows: lane1, untreated hMDMs; lane 2, hMDMs treated with 10 ng/mL LPS; lane 3, hMDMs treated with 10 ng/mL LPS and 10 μM NPS2143; lane 4, hMDMs treated with 10 ng/mL LPS and 1 μM latrunculin A.
